# Predicting drug polypharmacology from cell morphology readouts using variational autoencoder latent space arithmetic

**DOI:** 10.1371/journal.pcbi.1009888

**Published:** 2022-02-25

**Authors:** Yuen Ler Chow, Shantanu Singh, Anne E. Carpenter, Gregory P. Way

**Affiliations:** 1 Imaging Platform, Broad Institute of MIT and Harvard, Cambridge, Massachusetts, United States of America; 2 Brookline High School, Brookline, Massachusetts, United States of America; 3 Center for Health AI and Department of Biochemistry and Molecular Genetics, University of Colorado School of Medicine, Aurora, Colorado, United States of America; North Carolina State University, UNITED STATES

## Abstract

A variational autoencoder (VAE) is a machine learning algorithm, useful for generating a compressed and interpretable latent space. These representations have been generated from various biomedical data types and can be used to produce realistic-looking simulated data. However, standard vanilla VAEs suffer from entangled and uninformative latent spaces, which can be mitigated using other types of VAEs such as β-VAE and MMD-VAE. In this project, we evaluated the ability of VAEs to learn cell morphology characteristics derived from cell images. We trained and evaluated these three VAE variants—Vanilla VAE, β-VAE, and MMD-VAE—on cell morphology readouts and explored the generative capacity of each model to predict compound polypharmacology (the interactions of a drug with more than one target) using an approach called latent space arithmetic (LSA). To test the generalizability of the strategy, we also trained these VAEs using gene expression data of the same compound perturbations and found that gene expression provides complementary information. We found that the β-VAE and MMD-VAE disentangle morphology signals and reveal a more interpretable latent space. We reliably simulated morphology and gene expression readouts from certain compounds thereby predicting cell states perturbed with compounds of known polypharmacology. Inferring cell state for specific drug mechanisms could aid researchers in developing and identifying targeted therapeutics and categorizing off-target effects in the future.

## Author summary

We train machine learning algorithms to identify patterns of drug activity from cell morphology readouts. Known as variational autoencoders (VAE), these algorithms are unsupervised, meaning that they do not require any additional information other than the input data to learn. We train and systematically evaluate three different kinds of VAEs, each that learn different patterns, and we document performance and interpretability tradeoffs. In a comprehensive evaluation, we learn that an approach called latent space arithmetic (LSA) can predict cell states of compounds that interact with multiple targets and mechanisms, a well-known phenomenon known as drug polypharmacology. Importantly, we train other VAEs using a gene expression assay known as L1000 and compare performance to cell morphology VAEs. Gene expression and cell morphology are the two most common types of data for modeling cells. We discover that modeling cell morphology requires vastly different VAE parameters and architectures, and, importantly, that the data types are complementary; they predict polypharmacology of different compounds. Our benchmark and publicly available software will enable future VAE modeling improvements, and our drug polypharmacology predictions demonstrate that we can model potential off-target effects in drugs, which is an important step in drug discovery pipelines.

## 1. Introduction

A variational autoencoder (VAE) is a generative model that can generate realistic simulated data [1]. As an unsupervised model, a VAE is data-driven and learns by reconstructing input data rather than by minimizing classification error as in a traditional supervised neural network. A VAE compresses data into a lower-dimensional representation and then decodes it back into the original dimensions. The compressed lower dimensional space is often referred to as a “latent space”.

The so-called “vanilla” VAEs (i.e. VAEs as they were originally formulated [1]) minimizes the loss for the sum of reconstruction and Kullback–Leibler (KL) divergence. KL divergence is a regularization term that encourages the latent space to best approximate the data-generating function, which often improves model interpretation and data simulation. Vanilla VAEs are the standard choice of variational autoencoders and have provided an important foundation in the use of generative deep learning models. However, researchers have recently identified areas for improvement, and have thus modified VAE’s loss function to overcome these issues. For example, modifying the contribution of the KL divergence term encourages disentangled latent space features, as in β-VAE [2]. Another variant, the so-called InfoVAE or MMD-VAE, replaces the KL divergence term with maximum mean discrepancy (MMD) to improve the model’s ability to store more information in the latent space [3].

Recently, VAEs have been successfully trained on various biomedical data modalities such as bulk and single-cell gene expression data [4] from different assays measuring cell line perturbations and patient-derived tissue [5–8], DNA methylation [9], and cell image pixels [10,11]. β-VAEs have been used to produce disentangled latent representations of single cell RNA-seq data [12]. Similarly, MMD-VAEs have helped retain information in the latent space in single-cell data analysis of mass cytometry and RNAseq [13]. We trained vanilla-VAEs, β-VAEs, and MMD-VAEs only, and not other VAE variants and other generative model architectures, such as generative adversarial networks (GANs), because the three VAE variants we used are known to facilitate latent space interpretability.

One powerful application of VAEs and other generative models is the ability to simulate meaningful new samples using an approach called latent space arithmetic (LSA). A relatively simple approach, LSA uses a series of additions and subtractions of specific sample groups in their mean latent space representations to generate synthetic samples containing specific patterns captured via arithmetic. For example, LSA has been performed using a Deep Convolutional Generative Adversarial Network (DCGAN) model to generate new representations of faces, generating realistic but synthetic images in an LSA experiment: images of men with glasses—images of men without glasses + images of women without glasses = images of women with glasses [14]. Similarly, LSA using a generative model trained on fluorescent microscopy images, CytoGAN, predicted how cell images would look with increased nucleus size and increasing amounts of β-tubulin [15].

**Fig 1:**
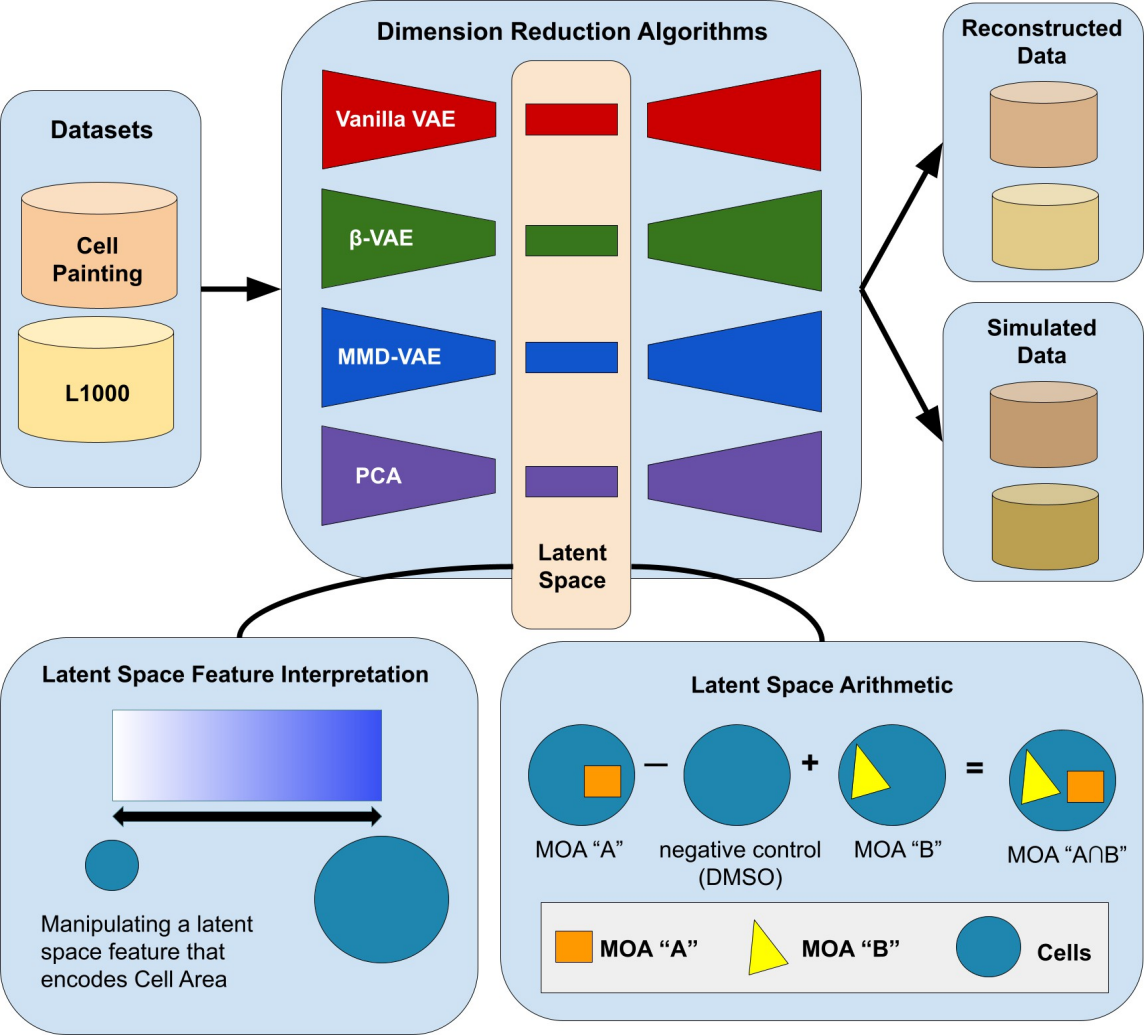
Our variational autoencoder (VAE) implementation framework, applied to determining the phenotype of cells. One application is to predict the phenotype of cells treated with compounds that have two mechanisms of actions (MOA), given the phenotype of cells treated with compounds that have each of those single MOAs (bottom right). A VAE encodes input data into a lower-dimensional latent space and then decodes the representation back into the original data dimensions. Our data contained measurements for 588 morphology features, each averaged for each population of cells treated with a given chemical compound. Following a sweep to select optimal hyperparameters (see Methods), we set our latent space dimension to 10 dimensions. The vanilla VAE learns by minimizing a reconstruction and KL-divergence loss. The other VAE variants we tested minimize loss functions that encourage disentangled features that promote interpretability, data simulation, and enable meaningful LSA.

Because of the success of VAEs on these various datasets, we sought to determine if VAEs could also be trained using cell morphology readouts (rather than directly on images), and further, to carry out arithmetic to predict novel treatment outcomes. We derive the cell morphology readouts using CellProfiler [16], which measures the size, structure, texture, and intensity of cells, and use these readouts to train all models. To see how VAE modeling ability compared across different data types, we also trained and evaluated VAEs on two other datasets: 1) the same cell morphology data but using all replicates instead of collapsed perturbation-specific signatures, and 2) gene expression data of the same perturbations. For our two cell morphology datasets, we used publicly-available cell morphology readouts from a Cell Painting experiment of 10,368 drug profiles from the Drug Repurposing Hub [17]. Our gene expression data comes from the same perturbations as measured by the L1000 assay, which quantifies mRNA transcript abundance of 978 landmark genes [18]. We trained a Vanilla VAE, β-VAE, MMD-VAE, and Principal Components Analysis (PCA) on each of these datasets to compare their performance in terms of reconstruction, latent space interpretability, and predicting polypharmacology representations from these datasets (**Fig 1**). Understanding the behavior of VAE architectures in modeling morphology representations will help us to interpret various signals of morphology systems biology. Characterizing drug MOAs is a crucial step to understanding how drugs work, and for monitoring and developing new therapeutics [19].

## 2. Results

### 2.1 Training variational autoencoders on cell morphology readouts

We trained our unsupervised learning models using data representing morphology readouts of A549 lung cancer cells treated with 1,571 compound perturbations from the Drug Repurposing Hub across 6 doses. Specifically, we used processed Cell Painting consensus signatures (level 5 data) from the Library of Integrated Network-Based Cellular Signatures (LINCS) [20]. Many of these drugs have annotations for their molecular targets and mechanisms of action (MOA). We split our input data into 80% training, 10% validation, and 10% test data balanced by plate and performed a Bayesian hyperparameter optimization using hyperopt [21].

Using optimal hyperparameters, we trained three types of VAEs: Vanilla VAE, β-VAE, and MMD-VAE and observed lower loss across epochs in real data compared to randomly permuted data (by independently shuffling the rows of each column, thus removing all correlation between features). This indicates that our VAEs have learned the data distribution by understanding the correlation between features because performance is worse when we remove correlation structure (**S1 Fig**). We observed similar trends with the Cell Painting replicate profiles (level 4 data) and the L1000 datasets (**S2 and S3 Figs**). We provide latent space embeddings for all profiles in our github repository https://github.com/broadinstitute/cell-painting-vae [22].

Each VAE variant learns by minimizing different loss functions, which provide different constraints and learn different latent space representations (see Methods for more details). When training the β-VAE, we observed that too high a β resulted in an uninformative latent space. The decoder did not fully utilize the latent code to reconstruct input samples. On the other hand, too low a β resulted in an entangled latent space, reducing the ability to interpret latent space features and perform the LSA experiment. So, we determined the optimal β by using a method that reduces the similarity between simulated and real data (see Methods). The ability to simulate data points requires a balance of reconstruction and disentanglement, hence finding the optimal value of β that results in the best simulation would likely improve performance in the LSA experiment.

**Fig 2:**
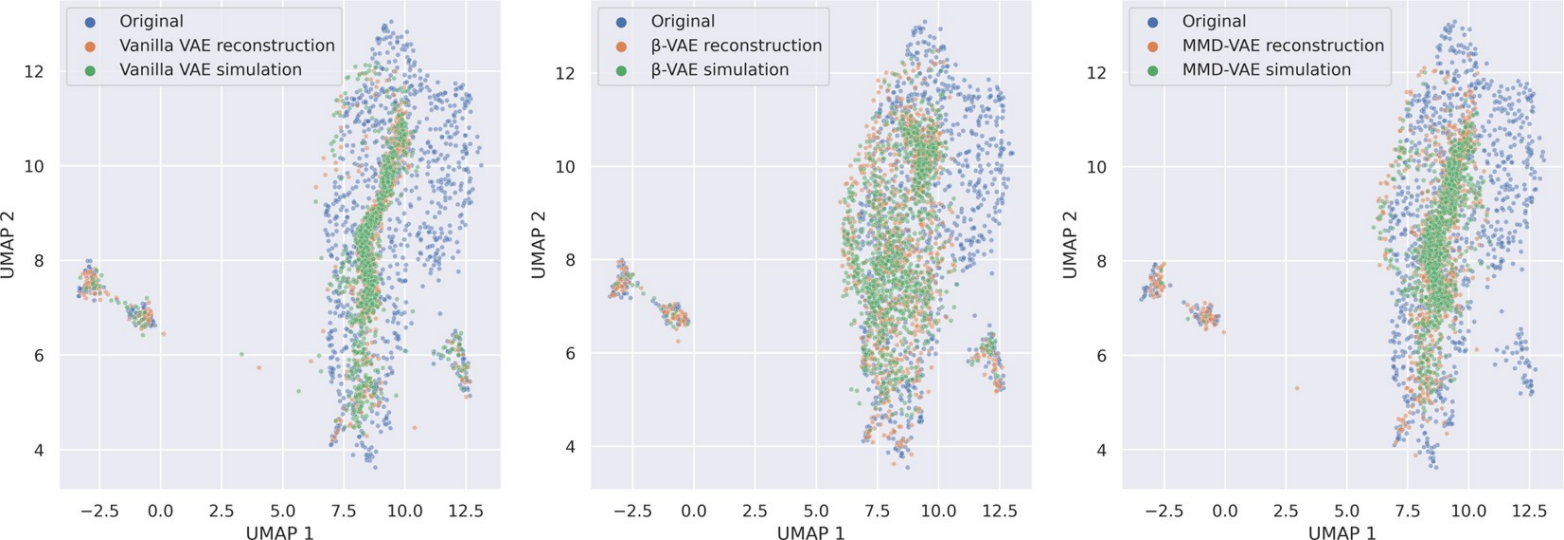
Two-dimensional UMAP embeddings of original, reconstruction, and simulated data for Cell Painting level 5 consensus signatures in the test set. We fit UMAP using only the original test set data and transformed the reconstructed and simulated data into this space. We simulated data by sampling from a unit Gaussian with the same dimensions as the latent space, using the same number of points as samples in the test set.

Next, we analyzed the ability of our trained VAEs to reconstruct individual samples and to simulate data. In two-dimensional Uniform Manifold Approximation and Projection (UMAP) [23] embeddings, we observed that real data overlapped with both reconstructed and simulated data indicating the ability of our models to reliably approximate the underlying morphology data generating function **(Fig 2)**. Both reconstructed and simulated data did not span the full original data distribution but were more constricted in the Vanilla-VAE compared to β and MMD-VAEs. Based on reconstruction loss (MSE) and earth moving distance, β-VAEs performed the best in Cell Painting data, and all architectures performed substantially better in most cases compared to randomly shuffled baselines **(Table 1)**. Our VAEs were also able to similarly reconstruct and simulate Cell Painting level 4 and L1000 data **(S4 Fig)**. We also observed a tradeoff between the VAEs ability to reconstruct samples and disentangle features, as indicated by improved reconstruction but higher latent space feature correlations in β-VAE compared to Vanilla VAE **(S5 Fig)**.

**Table 1:** Mean squared error (MSE) and earthmoving distance for VAE’s ability to reconstruct Cell Painting and L1000 profiles. We compare these values with results derived from shuffled models. Earthmoving distance is calculated by taking the mean of the earthmoving distance of each sample. We add the 95% percentile range of earthmoving distance in parenthesis (0.05 lowest, 0.95 highest). Note that since our models required that we normalize Cell Painting and L1000 input data differently (see Methods), the metrics cannot be compared across data modalities.

Dataset	VAE	MSE	MSE (Shuffled)	Earthmoving	Earthmoving (Shuffled)
Cell Painting level 5	Vanilla	0.00387	0.0088	0.016 (0.006, 0.040)	0.024 (0.0096, 0.060)
Cell Painting level 5	Beta	0.00272	0.0088	0.012 (0.005, 0.028)	0.024 (0.01, 0.06)
Cell Painting level 5	MMD	0.00435	0.0088	0.016 (0.005, 0.04)	0.024 (0.01, 0.06)
Cell Painting level 4	Vanilla	0.00145	0.0014	0.006 (0.004, 0.009)	0.0053 (0.004, 0.008)
Cell Painting level 4	Beta	0.00091	0.0014	0.0045 (0.002, 0.013)	0.0051 (0.004, 0.008)
Cell Painting level 4	MMD	0.00075	0.0014	0.0039 (0.0022, 0.01)	0.0051 (0.004, 0.008)
L1000	Vanilla	0.85	1.85	0.249 (0.14, 0.36)	0.61 (0.28, 1.94)
L1000	Beta	1.23	2.10	0.445 (0.23, 0.73)	0.67 (0.29, 2.25)
L1000	MMD	1.27	2.05	0.475 (0.25, 0.79)	0.64 (0.29, 2.027)

We also analyzed our VAE’s ability to reconstruct specific CellProfiler features. As expected, we observed that features with lower variance were more easily reconstructed **(S6 Fig)**. For all VAE variants, we found a high performance diversity, with many features reconstructing nearly perfectly, while others had poor reconstruction. The DNA channel was among the best reconstructed, while the AGP image channel was among the worst reconstructed, although this relationship was not significant (DNA < AGP with one-sample t-test p-value 0.37, 0.056, 0.20 for vanilla, β, and MMD-VAEs, respectively). The cells compartment was significantly better reconstructed compared to cytoplasm and nuclei for all VAE variants (Cells < non-Cells with one-sample t-test p-value 2.7e-4, 5.1e-7, 1.8e-5 for vanilla, β, and MMD-VAEs, respectively).

### 2.2 Interpreting cell painting latent space feature representations

As part of training, VAEs use different combinations of input features to generate representations. Specifically in our data, these so-called “representations” are, in essence, different combinations of morphology features that best capture signals from the input Cell Painting data. To facilitate interpretation and to understand the contribution of all morphology features to each latent space feature, we performed the following procedure: 1) Simulate +3 standard deviations of activity from one latent space feature while fixing all other latent features to zero, 2) Simulate -3 standard deviations of activity from the same latent space feature while fixing all other features to zero, 3) Pass both of these extreme-in-one-latent-feature latent spaces through the trained decoders, and 4) Subtract these two reconstructions from each other. Effectively, this procedure systematically implicates the most influential morphology features per latent space feature. This approach is similar to investigating specific VAE weight matrices (similar to PCA “loadings”) but it does not require us to set a threshold defining significant morphology feature contributions per latent feature.

As expected, we observed that our β-VAE learned more active latent features across a wide variety of morphology feature groups, compared to our Vanilla VAE and a baseline PCA. We also noticed that unlike the β-VAE where 5 of the 10 features encoded little information, all columns in our MMD-VAE were active, which indicates a more informative latent space that uses a wider diversity of morphology feature categories (**S7 Fig**).

**Fig 3:**
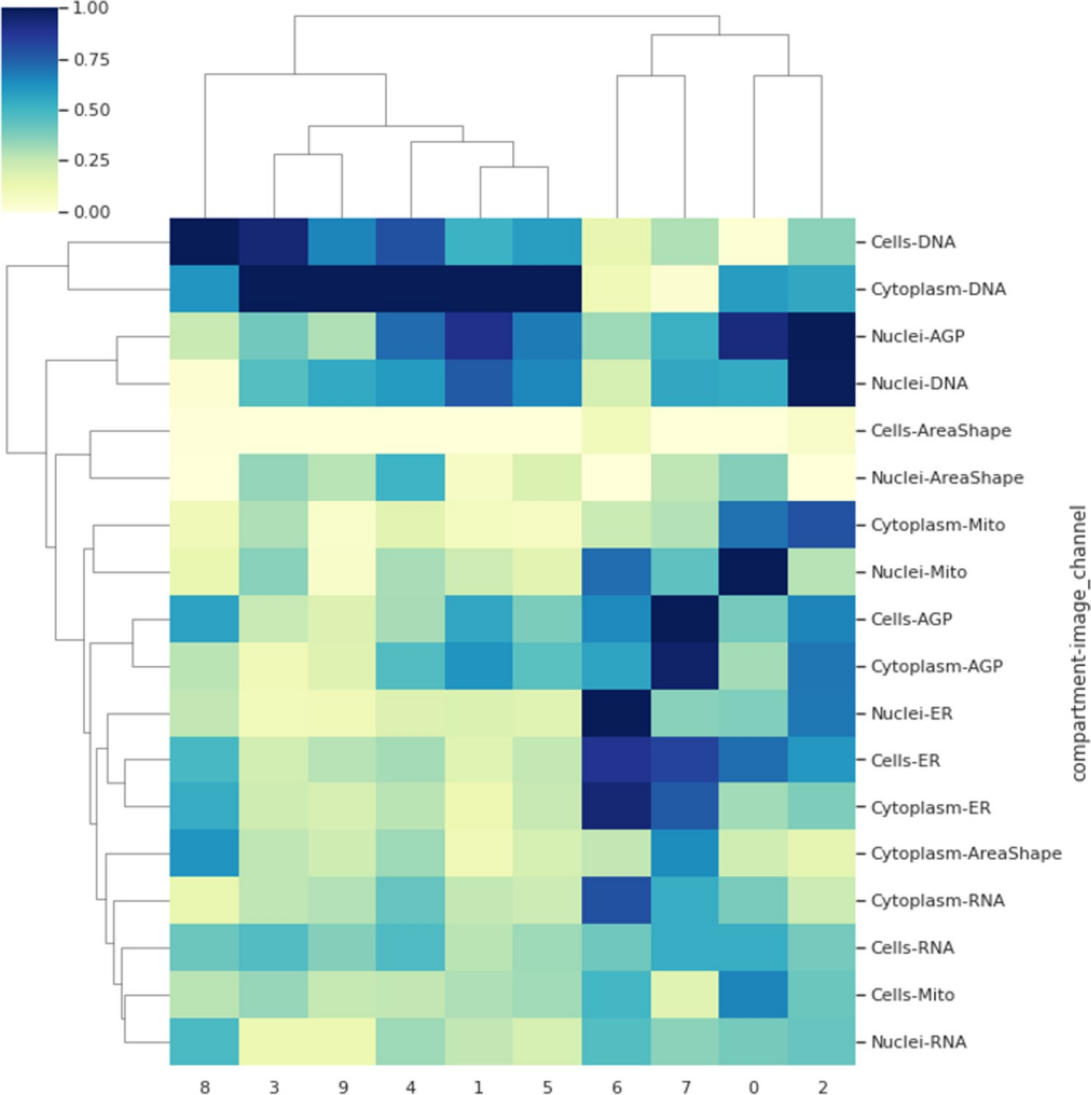
Investigating the contribution of CellProfiler feature groups (by compartments and image channels) on individual MMD-VAE latent space features. The dendrogram represents a hierarchical clustering algorithm applied to both rows and columns. Each color represents the mean contribution of each CellProfiler feature group to the given latent space feature normalized by column (see Methods for complete details).

Focusing on the MMD-VAE features, we observed that many individual latent space features encoded specific image channels and cell compartments (**Fig 3**). For example, latent feature 0 most strongly encoded Nuclei-Mito features (morphology features derived from the nucleus, specifically from the mitochondria fluorescent marker), feature 1 most strongly encoded Cytoplasm-DNA, and feature 2 most strongly encoded Nuclei-AGP and Nuclei-DNA features. The ability of the MMD-VAE to isolate these specific signals in an unbiased manner provides evidence that each image channel and compartment encodes unique information, and that these features can be used to interpret perturbation mechanisms in the future.

### 2.3 Predicting polypharmacological cell states with latent space arithmetic

The Drug Repurposing Hub has annotated the mechanism of action (MOA) of almost all compound perturbation profiles in the LINCS Cell Painting dataset [17,20]. The MOAs represent experimentally-derived classifications indicating the most likely biological mechanism(s) of the compound. Many compounds were annotated with a single MOA, but about 14% of these compounds (214 / 1570) were annotated with two MOAs (denoted using the form “A ∩ B”), indicating known polypharmacology; that there is evidence the compound acts through at least two separate mechanistic pathways, a common property even for marketed drugs [19,24]. In all, the Cell Painting dataset included 84 different MOAs.

We predicted that subtracting the mean DMSO from the mean MOA “A” in the latent space (“A”—“D”) would allow us to obtain the essential latent space information for a profile labeled with MOA “A”. Then, adding the mean representation of latent space values for profiles with MOA “B” would allow us to obtain a compressed representation for polypharmacology profiles labeled with MOA “A ∩ B”. We could then pass this latent representation through a VAE decoder to obtain a predicted cell with an “A ∩ B” cell state. Taken together, our latent space arithmetic equation hypothesis is “A”—“D” + “B” = “A ∩ B”. We performed these analyses using all the data, including training and test sets because our test set did not contain enough variety in samples to perform LSA.

**Fig 4:**
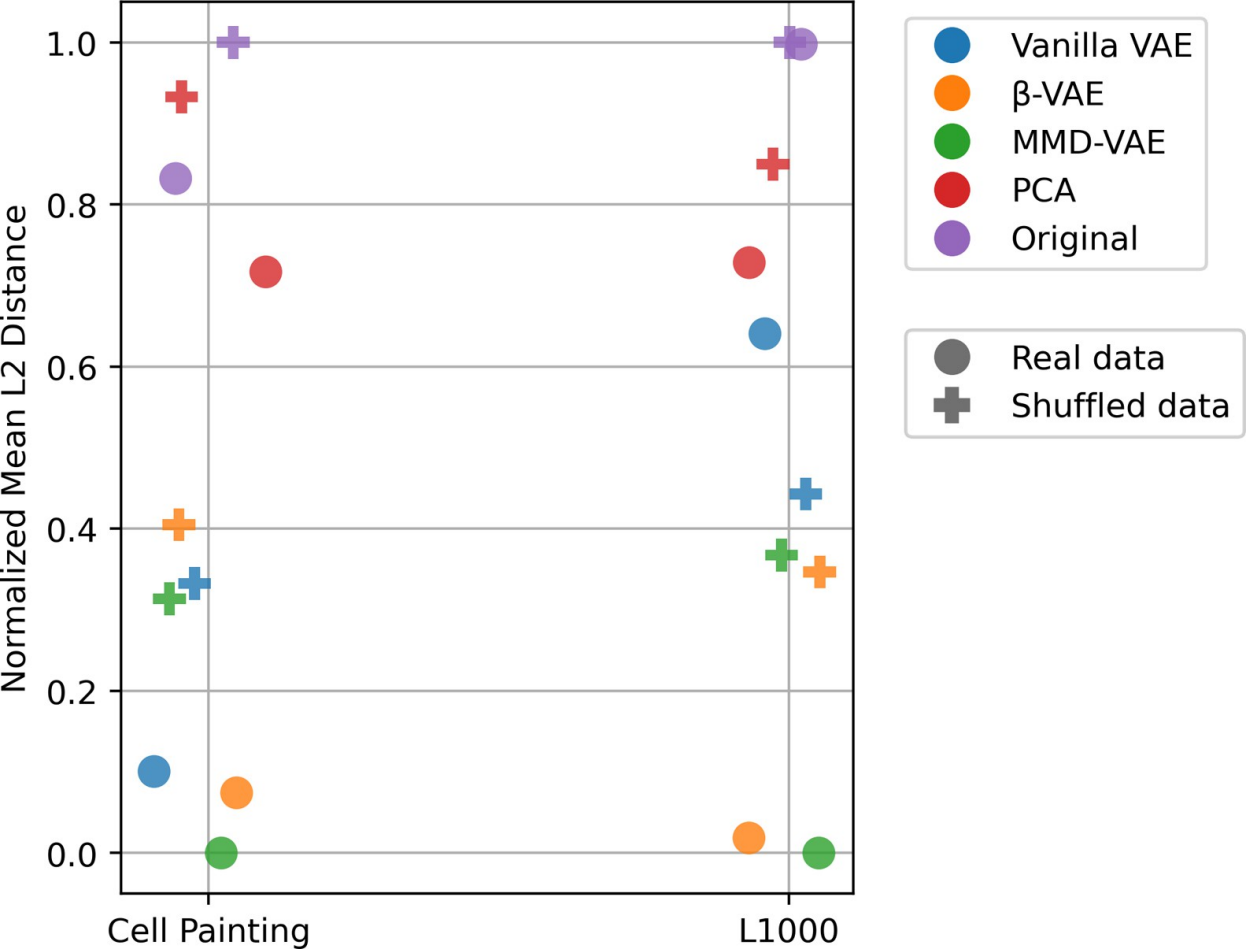
Mean L2 distance (lower is better) between real and predicted profiles annotated with known polypharmacology (“A ∩ B”) mechanisms of action (MOAs) for three different VAE architectures, PCA, and original input space. We show results for real and shuffled data across the two LINCS datasets. To enable a more meaningful and interpretable view, we zero-one normalized the L2 distances for each dataset. Each dot represents the mean L2 distance (values are normalized within each dataset) when LSA is performed using a specific model on a specific dataset.

To evaluate LSA performance, we calculated the L2 distances between the predicted and the actual “A ∩ B”. The mean L2 distance for VAEs with unshuffled MOAs was lower than shuffled data, lower than the original input dimensions, and lower than PCA, indicating that, on average, VAEs were better at predicting polypharmacological cell states **(Figs 4 and S8)**. Of the different VAE architectures, MMD-VAE performed the best in all datasets. We observed similar results when measuring MOA similarity using Pearson correlation **(S9 Fig)**. Importantly, we also observed improved predictability for MOAs that had a greater distance away from the mean Cell Painting feature values, indicating that MOAs are easier to predict when they have a more unique phenotype, further providing support for our ability to reveal true polypharmacology cell states rather than just predicting cell states with little to no phenotype **(S10 Fig)**.

### 2.4 Evaluating specific polypharmacology MOA predictions

**Fig 5:**
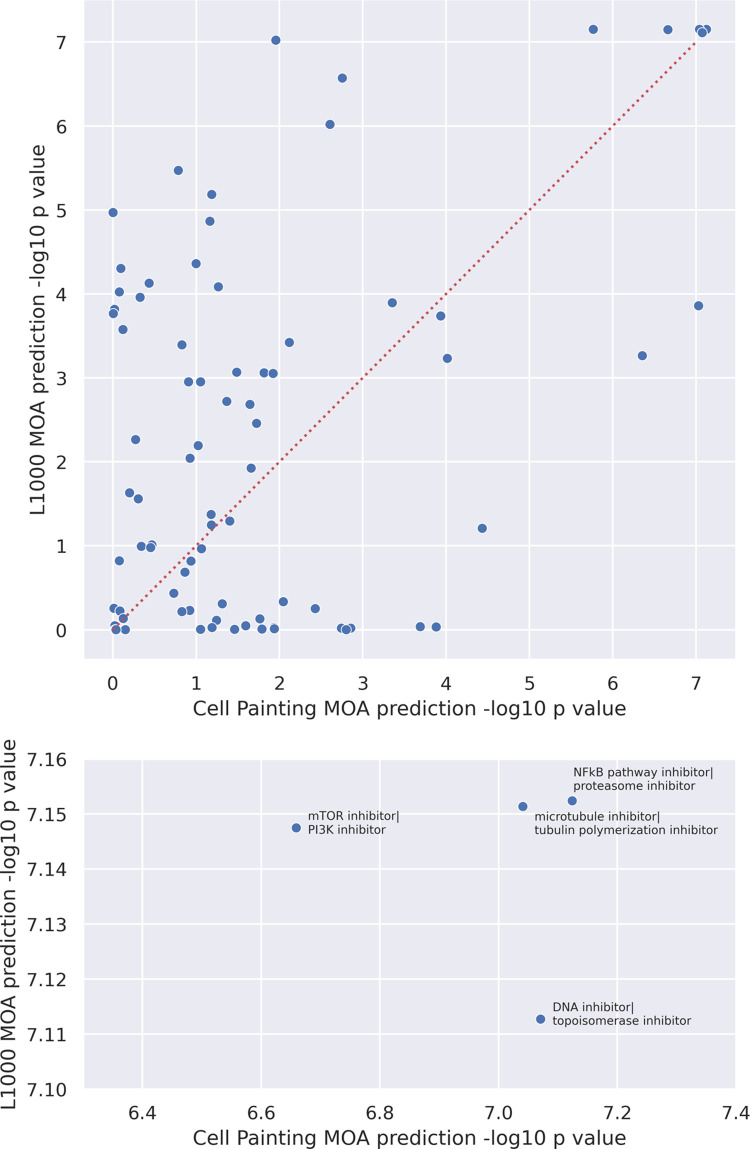
Scatterplot of L1000 vs Cell Painting MOA performance for MMD-VAE, with outliers (>3 stds) excluded. Performance is determined by the test scores between the L2 distance between predicted and real profile and the distribution of L2 distances from shuffling MOAs 10 times. *Top*—all “A ∩ B” combinations; *bottom*—only the top 5 “A ∩ B” with labels.

Using Cell Painting level 5 VAE models, we compared LSA performance of specific polypharmacology MOAs. For each polypharmacology MOA “A ∩ B”, we calculated a z score comparing 1) the L2 distance between the real and the predicted “A ∩ B” cell state against 2) a distribution of L2 distances between the real and ten predictions of “A ∩ B” cell states from randomly permuted input data (see Methods). We repeated this procedure using Pearson correlations as well. These metrics indicated that, for the majority of MOAs, we predicted polypharmacology states better than random (**S11 Fig**). High test statistics for L2 distance and a low test statistics for Pearson correlation indicates that the specific MOA “A ∩ B” could not be predicted, either because of incorrect annotations, non-additive or synergistic treatment effects, or a low penetrant phenotype unable to be captured in Cell Painting data. Compared to L1000 gene expression profiles, we observed that gene expression and morphology assays are complementary, but also predict many of the same polypharmacology MOAs **(Fig 5)**, which is consistent with recent work [25].

One limitation of our approach was that samples in our training data were annotated with many of the same MOAs as samples used in the LSA experiment, which could potentially leak information into our evaluation set and artificially inflate performance. For our work with predicting MOAs to have practical application, we needed to know that we can predict MOAs that have never been seen by the training set. So, we removed compounds annotated to the top five best-predicted MOAs, as well as the single MOAs that were part of the MOA combinations (i.e. also removing compounds annotated with MOA “A” or MOA “B” if MOA “A∩B” was in the top 5 best predicted) for each LSA evaluation. We then retrained VAEs for all VAE types. We observed that while sometimes the MOAs exhibited decreased performance, in general, those five MOAs remain among the best predicted out of all MOAs (**S12A Fig**). This reveals two things: First, it shows that the VAEs are stable; the same MOAs are still well-predicted when we retrain the model. Second, it shows that the VAEs do not need to have to have seen the specific cell states perturbed with compounds annotated with specific MOAs to predict their cell state and we are indeed not overfitting to the data.

We also retrained all models after removing compounds with MOA annotations that were predicted with intermediate performance. Specifically, we removed the five MOAs that were predicted with intermediate scores of above one standard deviation better than random. In general, performance dropped more for these intermediate data points than the high performers, but overall performance per MOA remained consistent particularly in our β-VAE (**S12B Fig)**. Despite the top MOAs retaining signal, lower performing MOAs experienced some signal loss, which suggests that intermediate performing MOAs are more susceptible to changes in training data composition and require more data or different approaches to model.

Finally, we performed two analyses to more specifically understand LSA performance. First, we assessed correlation between MOA reproducibility (reconstruction from the VAE) and MOA predictability (via the LSA experiment). We calculated MOA reproducibility by comparing median pairwise correlation between original and reconstructed “A∩B” MOAs, and we calculated MOA predictability as the correlation between real and predicted “A∩B” MOAs from the LSA experiment. We observed a strong correlation (Pearson r = 0.8) between MOA reproducibility and MOA predictability **(S13 Fig)** This was expected; MOAs that we more reproducibly captured are more reliably modeled and predicted. Second, we sought to see if there were any LSA performance differences depending on the type of MOA (inhibitor, antagonist, agonist, and other). While the top performing MOAs were all inhibitors, we did not observe significant differences across MOA types **(S14 Fig)**.

## 3. Discussion

Cell morphology provides an underexplored systems biology perspective of diseased and perturbed cell states. One current bottleneck in expanding this research avenue is the lack of generalizable, expressive, widely available, and interpretable morphology feature representations. Here, we used VAEs to model cell morphology representations of thousands of perturbed cell states. We determined that VAEs can be trained on cell morphology readouts rather than directly using the cell images from which they were derived. This decision comes with various trade-offs. Compared to cell images, cell morphology readouts as extracted by image analysis tools (e.g. CellProfiler) are a more manageable data type; the data are smaller, easier to distribute, substantially less expensive to analyze and store, and faster to train [16]. However, it is likely some biological information is lost because these tools might fail to measure all morphology signals. The so-called image-based profiling pipeline also loses information, by nature of aggregating inherently single-cell data to bulk consensus signatures [26]. Nevertheless, we successfully modeled cell morphology readouts from CellProfiler-derived representations of Cell Painting data using VAEs, and we demonstrated the power of these representations by simulating realistic-looking data, by deriving meaningful and disentangled morphology representations, and by predicting polypharmacology in certain compounds.

Using cell morphology readouts, we trained three different VAE architectures, each with different strengths and weaknesses. As expected, we observed improved modeling ability, as indicated by a lower reconstruction loss and improved data simulation, for a β-VAE model compared to a Vanilla VAE. Similarly, by using MMD-VAEs, which replaces the KL divergence term with one that calculates divergence for the full distribution of all latent features instead of each individual feature, we achieved better reconstruction than the vanilla VAE. However, the MMD-VAE failed to simulate all cell morphology modes, which the other VAE variants successfully captured. By training these VAE variants on L1000 gene expression readouts resulting from the same set of compound perturbations, we observed large differences in optimal hyperparameters as compared to training on Cell Painting image-based data [4,18]. These observations indicate that the KL divergence penalty strongly influences cell morphology modeling ability, and that lessons learned by modeling other biomedical data types, such as gene expression, will not necessarily directly translate to cell morphology [27].

Because we had access to the same perturbations with L1000 readouts, we were able to compare cell morphology and gene expression results. We found that both models capture complementary information when predicting polypharmacology, which is a similar observation to a deeper dive which compared the different technology information content [25]. We did not explore multi-modal data integration in this project, as this has been explored in more detail in other recent publications [28,29]. However, using multi-modal data integration with models like CycleGAN or other style transfer algorithms, might provide more confidence in our ability to predict polypharmacology in the future [30].

Through a deep inspection of the latent space features, we observed that the MMD-VAE learned the most informative representation (compared to vanilla and β-VAE), and, interestingly, uses information from all categories of Cell Painting readouts. The Cell Painting assay uses six different fluorescent stains to mark eight organelles: nuclei, endoplasmic reticulum, nucleoli, cytoplasmic RNA, actin, Golgi, plasma membrane, and mitochondria [31]. Subsequently, image analysts use software to segment cells to distinguish nuclei from cytoplasm and measure a broad set of hand-engineered, classical image features for each cell compartment across all five fluorescent channels [31]. While other emerging approaches for segmenting cells and extracting morphology features exist, some based on deep learning [32], the hand-engineered classical features in the profiles we used remain much more common and are currently the most directly interpretable. It remains uncertain if all Cell Painting fluorescent stains encode important, non-redundant information or if instead some could be acquired in simpler microscopy assays [33]. Here, we provide evidence that the different stains do encode independent biological signals, by nature of the unsupervised MMD-VAE disentangling these groups. However, our view of the biological signals embedded in the latent space representations is still limited; latent space features are likely encoding morphology signatures that extend beyond the individual feature categories we quantified (e.g. Cells-AGP), and represent higher order biological processes that are interacting between cell compartments and across image channels. Nevertheless, our optimal latent space dimension included only ten features, which is typically far fewer than other modalities such as gene expression [20,34,35].

Polypharmacology occurs when drugs interact with multiple targets, and it is a challenging aspect of drug discovery important for designing more effective and less toxic compounds [36]. Using established properties of generative models [14], we tested our three VAE variants in a so-called latent space arithmetic (LSA) experiment to predict polypharmacology cell states. Our results indicated that LSA worked best for the MMD-VAE architecture as compared to PCA and negative control baselines. MMD-VAEs allow for both disentanglement and a meaningful latent code, which supports LSA performance. While we observed only a slightly better LSA performance for all MOAs using randomly shuffled models compared to real data, there are several possible explanations. First, MOA annotations are often noisy, unreliable, and they change over time as scientists generate new knowledge about compounds [37]. However, the Drug Repurposing Hub MOA annotations are among the most well-documented resources, so other factors like different dose concentration and non-additive effects may also contribute to weak LSA performance for some compound combinations [17]. It is also possible that Cell Painting readouts may not capture certain MOAs that specifically manifest in other modalities. Indeed, we observed that certain polypharmacology target combinations were better predicted using gene expression readouts. Finally, we observed that LSA only performs well on certain MOA combinations, with most MOA combinations showing a negligible difference from its shuffled control. Therefore, while VAEs model interpretable latent spaces in Cell Painting data and LSA can serve as a baseline for predicting polypharmacology, other datasets, which directly collect data on an expanded set of polypharmacology cell states, will enable training of more accurate predictive models [38]. In the future, by predicting cell states of inferred polypharmacology, we can also infer toxicity using orthogonal models (e.g. [39]) and simulate the mechanisms of how two compounds might interact.

## 4. Conclusion

Cells are the building blocks of life, and they change when exposed to perturbations. There are many ways to measure, describe, and interpret how these cells change. In our analysis, we found that morphological cell states, as derived from microscopy images, can be modeled through VAE unsupervised learning to reveal biological insights. We found that several of our VAE models could reconstruct and simulate morphology data with high fidelity to ground truth perturbed cell states. When analyzing the latent code, we found that each latent space feature encoded different combinations of Cell Painting features. These feature representations were unique across different VAEs, with the MMD-VAE encoding the most information across Cell Painting channels and cell compartment feature groups. Our VAE models were able to simulate not only morphology but also gene expression cell states of compounds with multiple targets. Specifically, we simulated these polypharmacology cell states better than randomly shuffled and PCA controls. Several polypharmacology cell states performed better than others, with different performance for different MOA combinations for gene expression and morphology measurements. In the future, we could use unsupervised learning and mechanism predictions to interpret cell state mechanisms in different biological modalities, predict unknown MOAs, and characterize potential off-target effects in drug discovery and treatment. We provide all software, data, and results in an open source GitHub repository located at https://github.com/broadinstitute/cell-painting-vae [22].

## 5. Methods

### 5.1 Data acquisition

Previously, the Connectivity Map team at the Broad Institute of MIT and Harvard perturbed A549 cells with 1,515 different drug perturbations across six different doses as part of the Library of Integrated Network-Based Cellular Signatures (LINCS) consortium. They measured cell responses to these perturbations using the L1000 and Cell Painting profiling assays. We downloaded the publicly available LINCS Cell Painting dataset [20] and the publicly available LINCS L1000 data [18].

Briefly, Cell Painting is a fluorescent microscopy assay that uses a set of six unbiased dyes to mark DNA content, nucleoli, cytoplasmic RNA, endoplasmic reticulum (ER), actin, Golgi, plasma membrane, and mitochondria [31,40]. Briefly, L1000 is a bead-based gene expression assay that measures mRNA expression [18].

### 5.2 Data processing

For the Cell Painting assay, we previously applied an image analysis pipeline using CellProfiler. Previously, we used CellProfiler to segment and measure morphology features from single cells [16]. We then applied an image-based profiling pipeline, in which we aggregated and normalized single cells into compound profiles by dose per replicate [26]. We performed feature selection narrowing the initial 1,789 features to 584. We used four criteria for feature selection. We removed features with low variance, features that were blocklisted, features with missing values, and features with extreme outliers. Blocklisted features were those known to have caused issues in previous experiments and extreme outlier features were those that had a value greater than 15 standard deviations from the mean [41]. This procedure resulted in so-called “level 4” profiles. To form “level 5” consensus signatures, we collapsed level 4 replicate profiles into a single profile representing a compound-dose signature. For complete processing details, refer to https://github.com/broadinstitute/lincs-cell-painting. We further processed the Cell Painting data by performing 0–1 normalization on Cell Painting because not all features were on the same scale and we did not want different scales to influence the predictive power of a certain feature.

For L1000, we use the previously processed 978 “landmark” genes as our input features. We do not include all inferred genes because this would likely overload our VAE with redundant information. See [18] for complete processing details.

As input into our machine learning models, we split the data into an 80% training, 10% validation, and 10% test set, stratified by plate for Cell Painting and stratified by cell line for L1000. In effect, this procedure evenly distributes compounds and MOAs across data splits.

### 5.3 Variational autoencoder implementations

A standard Vanilla VAE [1] minimizes the loss for the sum of two loss functions: reconstruction (by mean squared error (MSE)) and Kullback–Leibler (KL) divergence. KL divergence encourages the latent space samples to follow a multivariate Gaussian distribution.


$$E_{q_{\phi }(z|x)}[\log\;p_{\theta }(x|z)]-KL(q(z|x)\|p(z))$$

In a β-VAE, we multiply the KL divergence term by a constant β [2]. The purpose of this is to achieve greater disentanglement in the latent code. Our method of determining the value of β is described in the next section “5.4 Training procedure”.


$$E_{q_{\phi }(z|x)}[\log\;p_{\theta }(x|z)]-\beta KL(q(z|x)\|p(z))$$

However, β-VAEs still suffer from two main problems. First is the “information preference property”, where if the KL divergence term is too high, all *z* values will be close to the prior p(x), leading to little useful information encoded in the latent space. The second problem is that if the regularization term is not strong enough, we get so-called “entangled” latent representations. These tradeoffs in a β-VAE led to the development of the maximum mean discrepancy (MMD)-VAE, where the scientists completely replaced the KL divergence term with a term that minimizes the mean maximum discrepancy [3]. Specifically, MMD forces the aggregated *z* distribution towards the prior rather than each individual *z* (as in the vanilla and β-VAE), which allows individual *z* values to diverge from the prior and more flexibly encode information [42]. Also, just like how we use β to adjust the magnitude of the KL divergence term, we use λ to adjust the magnitude of the MMD regularization term for our MMD-VAE. We calculated MMD efficiently using the kernel embedding trick [43].


$$E_{q_{\phi }(z|x)}[\log\;p_{\theta }(x|z)]-\lambda MMD(q_{\theta }(z)\|p(z))$$

We used an Adam optimizer for all VAE architectures and datasets, and we used a leaky rectified linear activation function (Leaky ReLU) for all intermediate layers. Each model was a two-layer VAE, meaning that both encoder and decoder had one hidden layer. This hidden layer had 250 nodes for Cell Painting and 500 nodes for L1000 (determined by approximately one half of the number of input features).

### 5.4 Determining β in our β-VAE

The original β-VAE paper explains how to choose β. It states that if we have labelled data, we should use a disentanglement metric. But in our case where our data is not labelled, “the optimal value of β can be found through visual inspection of what effect the traversal of each single latent unit z has on the generated images (x|z) in pixel space.” But this also didn’t apply to our situation since we were not using images.

So, we proposed a new method of determining β by measuring the similarity between the original training set and simulated data points by sampling from a uniform distribution as is traditional in VAE generative models. To simulate data points that are close to the original distribution of the data, the VAE needs to have a balance of reconstruction and disentanglement. It needs good reconstruction because we want simulated data to look like real data. But we also need disentanglement because we simulate data points by sampling from the prior multivariate Gaussian, so the VAE benefits from the KL divergence term that pushes *z* values closer to that distribution.

It is likely that this method is not perfect and has a bias towards a lower-than-optimal β. This is because while simulating data benefits from a disentangled latent space, as long as the latent code roughly aligns with the prior, the simulation will be decent. On the other hand, performing LSA also requires that each feature be independent of each other; we need to be able to independently adjust the features to generate new unseen profiles. Because of this, the β determined by this method is likely to be lower than what would be optimal to perform LSA. Nonetheless, using this method would still give us a lot better β than if we were to choose randomly. Additionally, in practice, we observed that different β had a relatively little effect on cross validation performance.

Specifically, in our β-optimizing method, we measured similarity by calculating the Hausdorff distance between the original training data and the same number of simulated data points [44]. Because simulating data points requires a balance of reconstruction and disentanglement—which is controlled by β—if we were to train the model many times with different values of β, we would observe that an intermediate β would result in the lowest Hausdorff distance. For L1000, β = 40 was optimal. We found that for our Cell Painting datasets (level 4 replicate profiles and level 5 consensus signatures), a β < 1 was optimal (0.3 for Cell Painting level 5 and 0.06 for level 4). So, in this way, our implementation of β-VAE for Cell Painting differs from the original concept of β-VAE where you would increase β > 1to achieve disentanglement. For all three datasets, the magnitude of the KL divergence term (after multiplied by β) was between 10–30% of the total loss (MSE + KLD) (CP5 0.79/2.37 = 33%, CP4: 0.12/0.76 = 15%, L1000: 133/1330 = 10%).

We first chose a reasonable set of hyperparameters to use based on our initial training observations (latent_dim = 50, learning_rate = 0.001, encoder_batch_norm = True, batch_size = 128, epochs = 50) to keep constant and then adjusted β across many training sessions. After determining the optimal β (lowest Hausdorff distance), we used this value as a constant during a hyperparameter sweep to determine the optimal value for the other hyperparameters.

We used the same hyperparameters to train both our Vanilla VAE and MMD-VAE. This was because the data would remain the same between different VAE variants, it is likely that the same hyperparameters would perform well as well. Also, because hyperparameters can have a big impact on training performance, keeping them consistent would be the best way to compare these models. Furthermore, we did not observe much difference in cross validation performance for many combinations of hyperparameters we tested. When training the MMD-VAE, we first tried using the Hausdorff distance method to find the optimal value for λ. But, increasing λ didn’t have much effect on simulated data even as we increased λ much greater than1. So, we decided to choose λ merely by choosing a large value that still resulted in a stable training curve. This was 1000 for Cell Painting level 5, 10,000 for Cell Painting level 4, and 10,000,000 for L1000. For all three datasets, the magnitude of the MMD term (after multiplied by λ) was between 74–98% of the total loss (MSE + MMD) (CP5: 11.07/14.99 = 74%, CP4: 14.39/14.75 = 98%, L1000: 1948/2514 = 77%). This proportion of the regularization term is a lot higher than it was in the β-VAE. This ability for us to increase the magnitude of the regularization in a MMD-VAE with little negative consequences is a property of MMD-VAEs because their loss function allows them to encode information in their latent space even when the regularization term is high.

### 5.5 Hyperparameter optimization

Using Keras Tuner, we performed Bayesian hyperparameter optimization on all three datasets to select optimal learning rate (1e-2, 1e-3, 1e-4, 1e-5), batch size (32 to 512, with increments of 32), latent dimension (5 to 150, with increments of 5), and encoder batch normalization (True, False) for a two-layer VAE **(Table 2 and S15 Fig).** The optimal latent dimension of 10 for Cell Painting level 5 data was surprising as it implies that 10 features are sufficient to encode Cell Painting profiles.

**Table 2:** Hyperparameter combination of the top performing models for each dataset.

Dataset	latent_dim	learning_rate	encoder_batch_ norm	batch_size	val_loss
Cell Painting level 5	10	0.001	True	96	2.24
Cell Painting level 4	90	0.0001	True	32	0.74
L1000	65	0.001	True	512	1363.45

### 5.5 Cell painting latent space feature interpretation

The optimal Cell Painting level 5 VAE models had 10 latent space features. Keeping all other latent space features at 0, we manipulated one feature at a time, comparing the reconstruction of +3 standard deviations with -3 standard deviations. Since σ of p(x) is 1, we used the values 3 and -3 for each latent space feature. To compare reconstructions, we took the absolute value of the difference between the two reconstructions. This output represents which original Cell Painting features most contribute to reconstruction of the single latent space feature. We repeated this procedure with all 10 latent space features.

### 5.6 Latent space arithmetic (LSA) approach to predict polypharmacology cell states

We transformed all 50,303 level 4 and 10,368 level 5 consensus profiles for the Cell Painting dataset, and 118,050 L1000 profiles into the latent space and then grouped them by their MOA annotation. We used the Drug Repurposing Hub MOA annotations [17]. To perform LSA, we first needed to filter compounds to include only those compatible with our hypothesis. Specifically, we included only compounds that satisfied the following rules. We only kept compounds with one or two MOA annotations. Also, for groups with only one annotated MOA, we only kept it if it corresponded to at least one group of compounds annotated with two MOAs. That is, if there was a group with MOA “A”, we only kept that group if there was another group with MOA “A ∩ B” or “A ∩ B”. The “∩” notation indicates that the compound has evidence for both mechanisms. We also kept the DMSO profiles (negative control), which lacks MOA annotations. We needed to keep this group because it was part of our LSA equation hypothesis (“A”—“D” + “B” = “A ∩ B”).

For each group, we calculated the mean for each latent space feature, giving us a vector of length 10 for each MOA combination. We included all doses of all annotated compounds to compute the mean latent space features. Then, for each existing “A ∩ B” in our data, we performed vector addition and subtraction on the groups of MOA “A”, “B”, and “D” using our LSA equation, allowing us to have a predicted latent space representation of “A ∩ B”. We then decoded this prediction to a reconstructed representation of the “A ∩ B” and compared this representation with the original, real-data representation. The original representation for an MOA was achieved by taking the mean value of profiles with that MOA for all features. This comparison was done by computing the L2 distance or Pearson correlation for each MOA combination (see results).

To determine if the LSA approach was significantly different from a randomly shuffled control, we randomly shuffled all MOA labels, including DMSO labels, and performed the same LSA experiment. This results in a second distribution of L2 distances between the mean profile of an actual MOA label and a predicted profile from performing LSA on random profiles. We performed this shuffling 10 times to get a representative distribution of random predictions, so our control distribution was 10 times larger than the unshuffled distribution.

To compare against negative control baselines, we performed this same LSA procedure using principal component analysis (PCA) with 10 principal components. We also performed LSA using the original data dimensions, without any dimensionality reduction.

To determine the MOAs our VAE could predict the best, we calculated a z-score metric for each MOA combination. Specifically, for a given polypharmacology MOA “A ∩ B”, we compared two values: 1) the L2 distance between the predicted cell state and the ground-truth cell state and 2) the distribution of L2 distances between each of the ten cell state predictions from the randomly shuffled negative control and the ground-truth cell state. As well, we repeated this procedure using Pearson correlation instead of L2 distance. This evaluation measures performance for each polypharmacology MOA “A ∩ B” independently and describes how much better LSA with real data could predict cell states compared to random. In addition to calculating z-scores, we also calculated p-values for Cell Painting and L1000 applying one-sample z-tests to the ground truth and randomly permuted distribution.

### 5.7 Computational reproducibility

All scripts and computational environments to download and process data, train all VAEs, and reproduce all results in this paper can be found at https://github.com/broadinstitute/cell-painting-vae [22].

## Supporting information

S1 Fig Training curves for three VAE models trained using Cell Painting level 5 data (replicate profiles). We show training and validation curves for real and shuffled data in three VAE variants: (a) Vanilla VAE, (b) β-VAE, and (c) MMD-VAE. The MMD-VAE training curve for shuffled data shows that it’s unstable. We believe a big reason for this is because of the fact that the optimal MMD-VAE had a much higher regularization term, which puts a greater emphasis on forming normal latent distributions, than the optimal Beta or Vanilla VAE. Forcing the VAE to consistently encode a shuffled distribution into a normally distributed latent distribution would be difficult, and therefore might cause oscillations in the training curve across epochs.
(TIFF)

S2 Fig Training curves for three VAE models trained using Cell Painting level 4 data (replicate profiles). We show training and validation curves for real and shuffled data in three VAE variants: (a) Vanilla VAE, (b) β-VAE, and (c) MMD-VAE.
(TIFF)

S3 Fig Training curves for three VAE models trained using L1000 level 5 data (replicate profiles). We show training and validation curves for real and shuffled data in three VAE variants: (a) Vanilla VAE, (b) β-VAE, and (c) MMD-VAE.
(TIFF)

S4 Fig **Two-dimensional UMAP embeddings of original, reconstruction, and simulated data for [A] Cell Painting level 4 replicate profiles and [B] L1000 level 5 consensus profiles in the test set.** We fit UMAP using only the original test set data and transformed the reconstructed and simulated data into this space. We simulated data by sampling from a unit Gaussian with the same dimensions as the latent space. We simulated the same number of points as samples in the test set.
(TIFF)

S5 Fig Latent space feature pairwise correlation heatmaps for VAE trained on Cell Painting level 5 consensus signatures. Lower correlation is an indication of disentanglement in the latent space. We show heatmaps for all three VAE variants: Vanilla VAE, β-VAE, and MMD-VAE.
(TIFF)

S6 Fig Comparing feature variance with feature reconstruction for Cell Painting level 5 input data across three VAE variants. We stratified feature categories by image channel and compartment.
(TIFF)

S7 Fig Cell morphology VAE latent space contributions by CellProfiler features. In the training process, VAEs learn latent space embeddings that represent the input features. Here, we systematically modify each latent space feature in isolation (by simulating each latent feature +/- 3 standard deviations, passing through the decoder, and subtracting the original data; see methods for complete details). Each square represents the mean difference between the reconstructed extreme latent feature simulations. The CellProfiler features represent three different cell compartments by five imaging channels plus AreaShape features. Each latent feature across VAE architectures captures a different combination of CellProfiler features. We show heatmaps for all three VAE variants (Vanilla VAE, β-VAE, and MMD-VAE) and the first 10 components of the PCA model.
(TIFF)

S8 Fig Full L2 distance distributions of latent space arithmetic to predict polypharmacology. We performed latent space arithmetic to predict cell states of polypharmacological compounds. This allowed us to calculate a L2 distance between predicted and real corresponding to each MOA for both shuffled and real data. We generated distributions for all three datasets and all three VAE variants.
(TIFF)

S9 Fig Correlation between real and predictions. Mean Pearson correlation (higher is better) between real and predicted profiles annotated with known polypharmacology (“A ∩ B”) mechanisms of action (MOAs) for three different VAE architectures, PCA, and original input space. We used level 5 Cell Painting input data for LSA predictions.
(TIFF)

S10 Fig MOA predictability compared to feature distance. Scatter Plot to visualize the relationship between MOA predictability (- log p value) and the distance between that MOA and the mean Cell Painting feature values in Cell Painting level 5 MMD-VAE. Higher values on the Y axis indicate better predictability, and higher values on the X axis indicate L2 distances to the mean of all profiles together.
(TIFF)

S11 Fig Distribution of LSA z-scores from comparing ground-truth MOA cell state to 10 randomly shuffled LSA permutations for Cell Painting level 5 data. The blue line is centered at 0. All MOAs to the left of the blue line for the L2 distance graph are predicted better than random, and all MOAs to the right of the blue line in the Pearson correlation graph are predicted better than random. The red line indicates the mean of all the z-scores, so a lower mean for the L2 distance is better, and higher mean for Pearson correlation is better.
(TIFF)

S12 Fig Leave-five-out MOA experiment demonstrates robust learning in the absence of certain compounds. Original polypharmacology MOA prediction latent space performance compared to performance after retraining the VAEs with the top five MOA combinations left out. The axes represent -log10 p value of the L2 distances between real polypharmacology cell states and shuffled cell states in the LSA experiment. The red points represent polypharmacology MOAs that we left out from the models on the y axis.
(TIFF)

S13 Fig MOA reproducibility and predictability. Strong correlation between MOA reproducibility (median pairwise correlation among real and reconstructed MOAs) and MOA predictability (correlation between real and predicted MOA from LSA experiment).
(TIFF)

S14 Fig No association between MOA compound class and the predictability of MOAs. (TIFF)

S15 Fig Visualizing the results of hyperparameter optimization for a two-layer VAE trained on Cell Painting level 5 data using Hiplot. Each line represents a single hyperparameter combination. We show training and validation loss in the last two columns, respectively.
(TIFF)
