# Supplementary figures and images for: Predicting drug polypharmacology from cell morphology readouts using variational autoencoder latent space arithmetic
Source: PLoS Comput Biol. 2022 Feb 25;18(2):e1009888. doi: 10.1371/journal.pcbi.1009888 (PMC8906577; doi:10.1371/journal.pcbi.1009888)

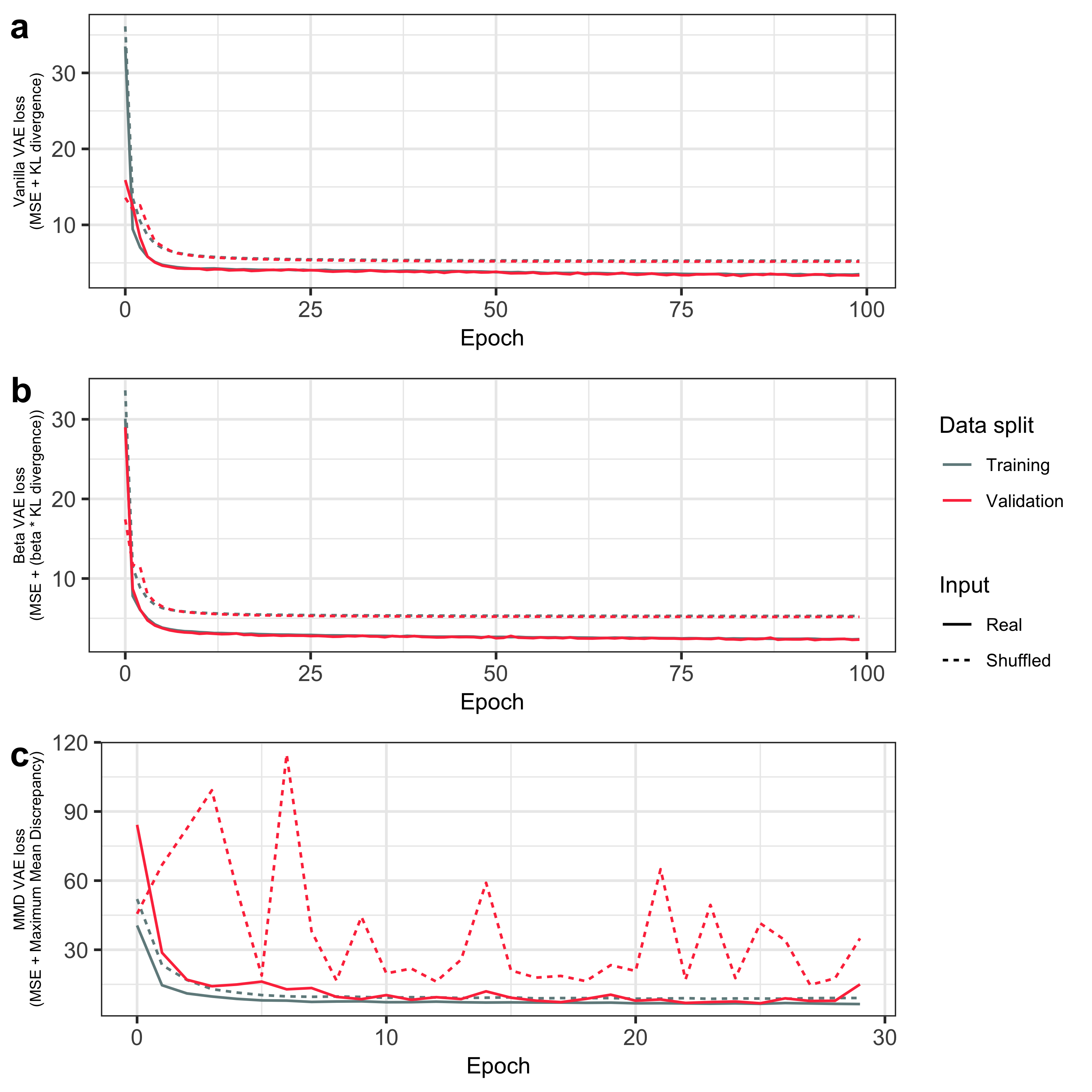

Supplement: S1 Fig — We show training and validation curves for real and shuffled data in three VAE variants: (a) Vanilla VAE, (b) β-VAE, and (c) MMD-VAE. The MMD-VAE training curve for shuffled data shows that it’s unstable. We believe a big reason for this is because of the fact that the optimal MMD-VAE had a much higher regularization term, which puts a greater emphasis on forming normal latent distributions, than the optimal Beta or Vanilla VAE. Forcing the VAE to consistently encode a shuffled distribution into a normally distributed latent distribution would be difficult, and therefore might cause oscillations in the training curve across epochs. (TIFF) [file pcbi.1009888.s001.tiff]

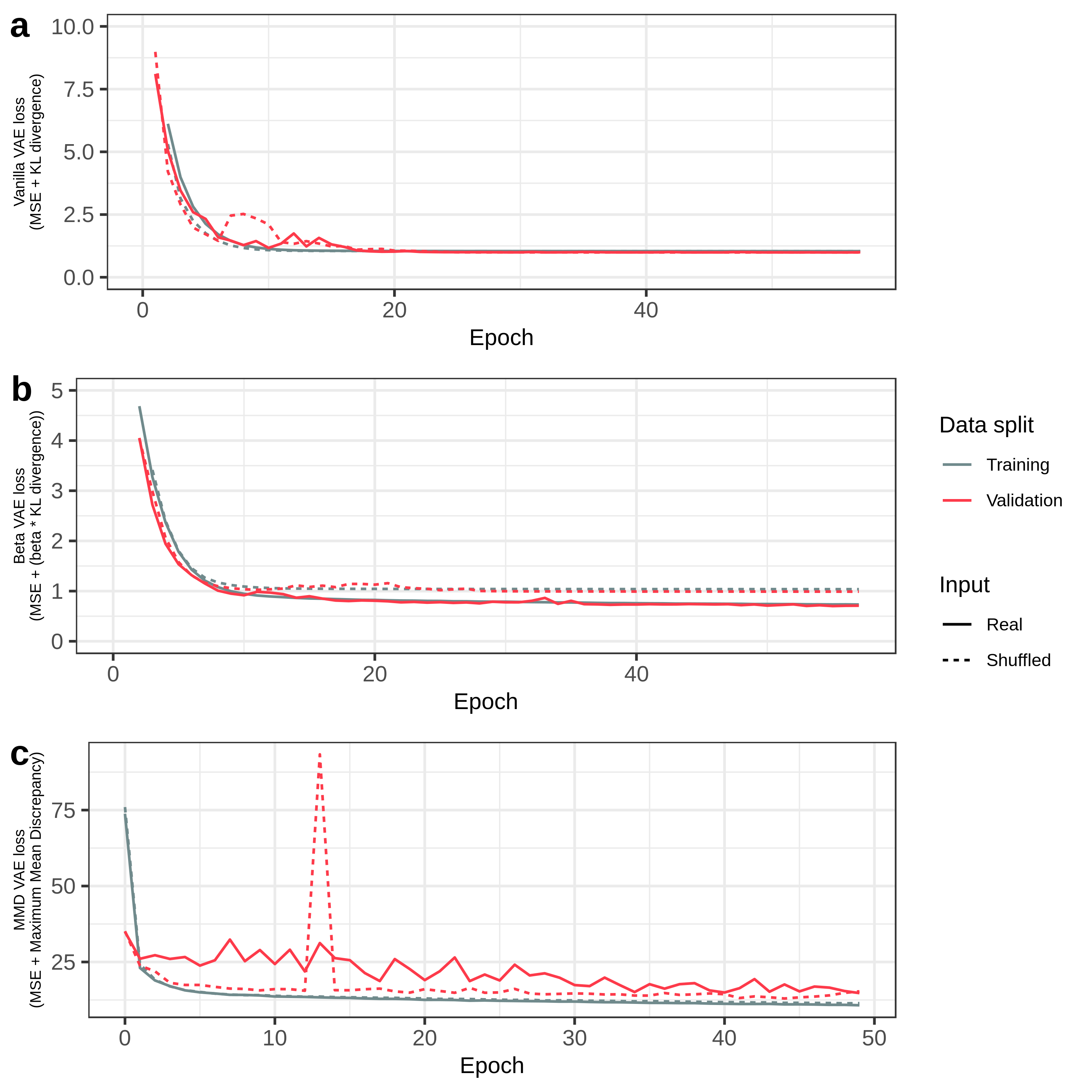

Supplement: S2 Fig — We show training and validation curves for real and shuffled data in three VAE variants: (a) Vanilla VAE, (b) β-VAE, and (c) MMD-VAE. (TIFF) [file pcbi.1009888.s002.tiff]

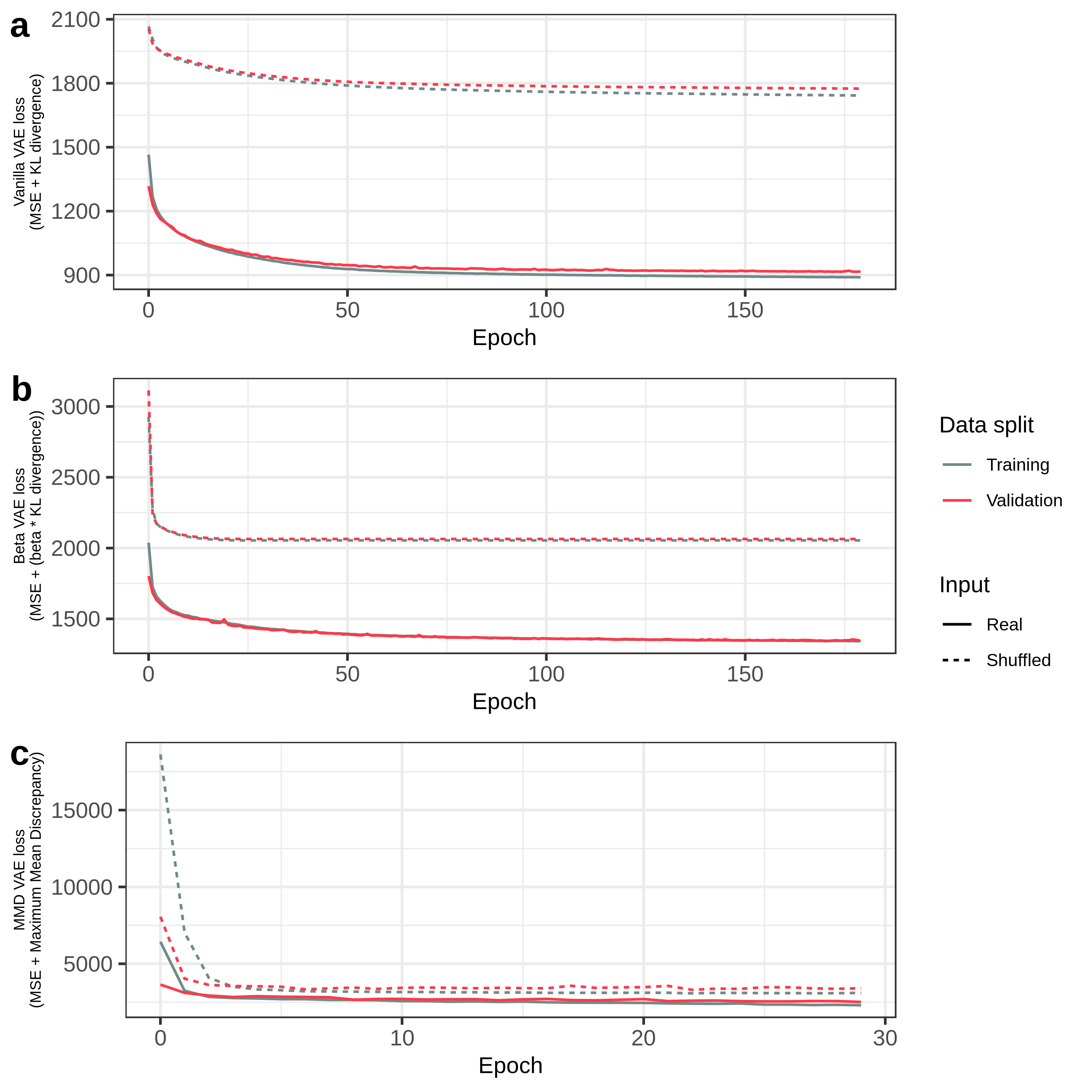

Supplement: S3 Fig — We show training and validation curves for real and shuffled data in three VAE variants: (a) Vanilla VAE, (b) β-VAE, and (c) MMD-VAE. (TIFF) [file pcbi.1009888.s003.tiff]

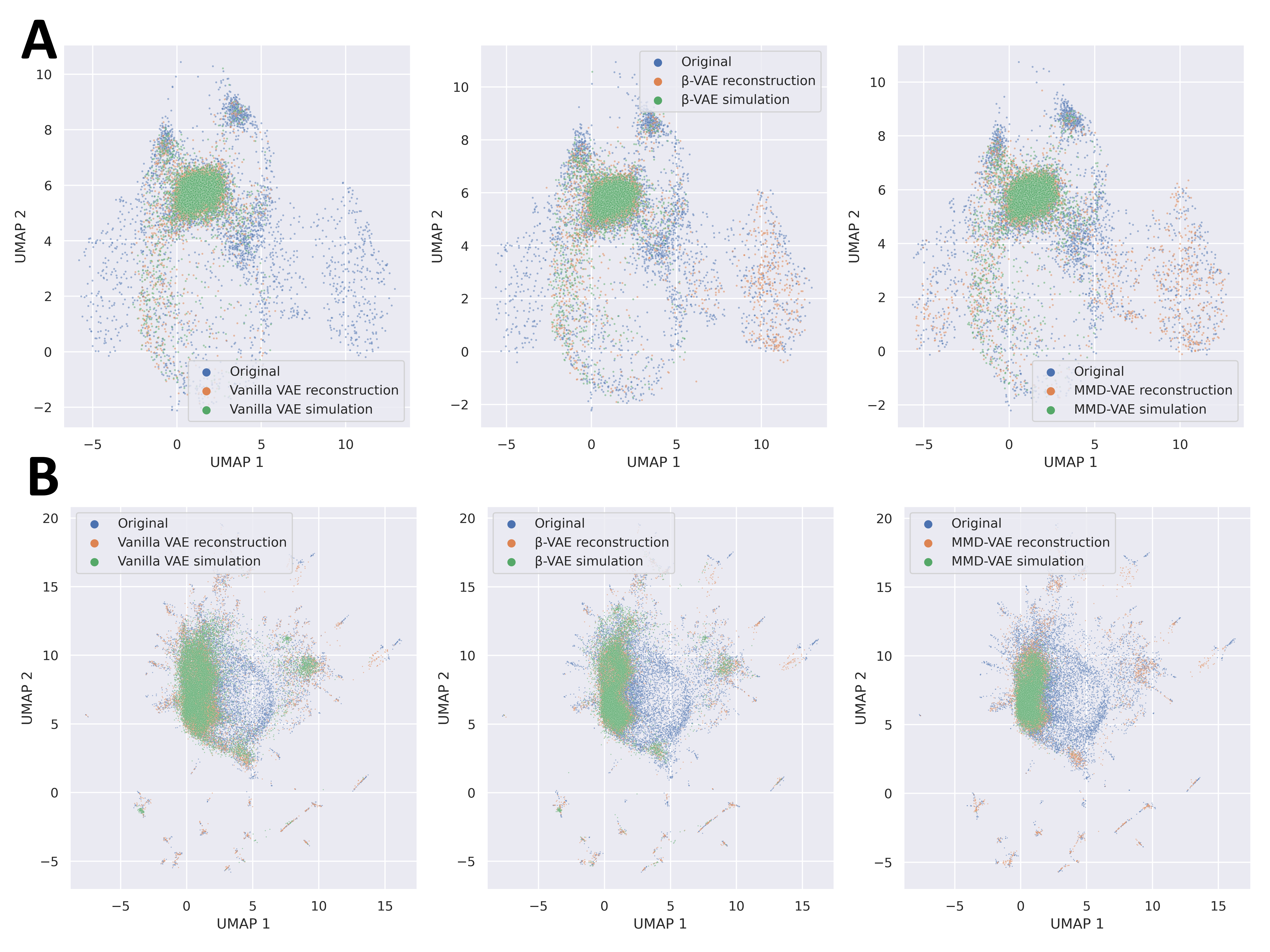

Supplement: S4 Fig — Two-dimensional UMAP embeddings of original, reconstruction, and simulated data for [A] Cell Painting level 4 replicate profiles and [B] L1000 level 5 consensus profiles in the test set. We fit UMAP using only the original test set data and transformed the reconstructed and simulated data into this space. We simulated data by sampling from a unit Gaussian with the same dimensions as the latent space. We simulated the same number of points as samples in the test set. (TIFF) [file pcbi.1009888.s004.tiff]

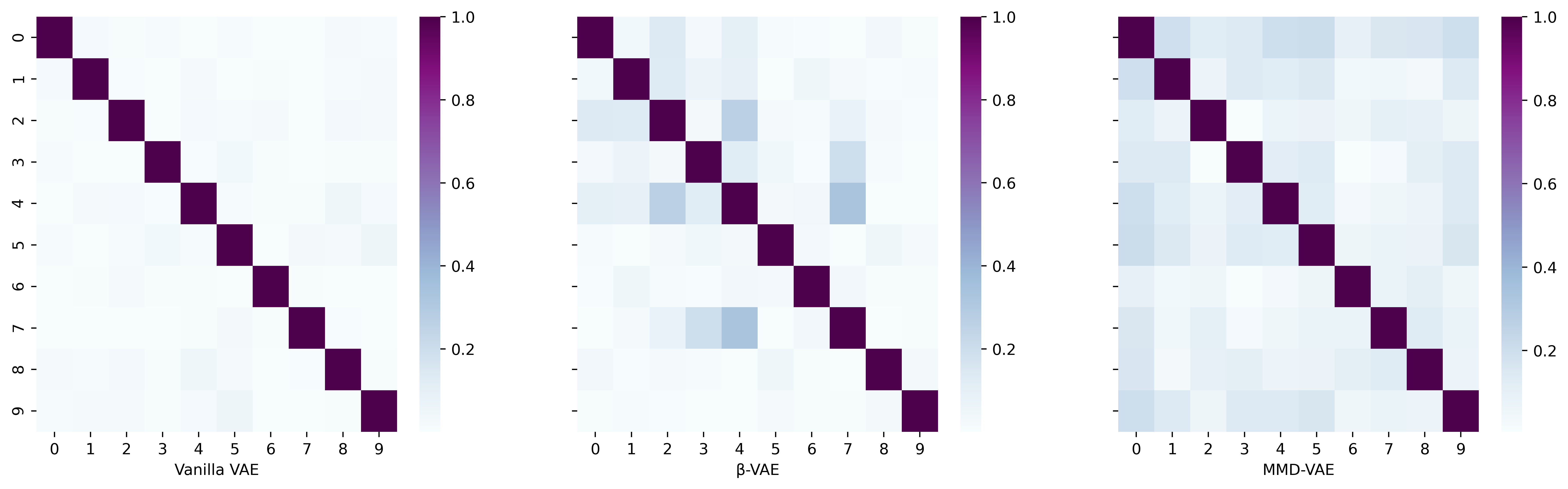

Supplement: S5 Fig — Lower correlation is an indication of disentanglement in the latent space. We show heatmaps for all three VAE variants: Vanilla VAE, β-VAE, and MMD-VAE. (TIFF) [file pcbi.1009888.s005.tiff]

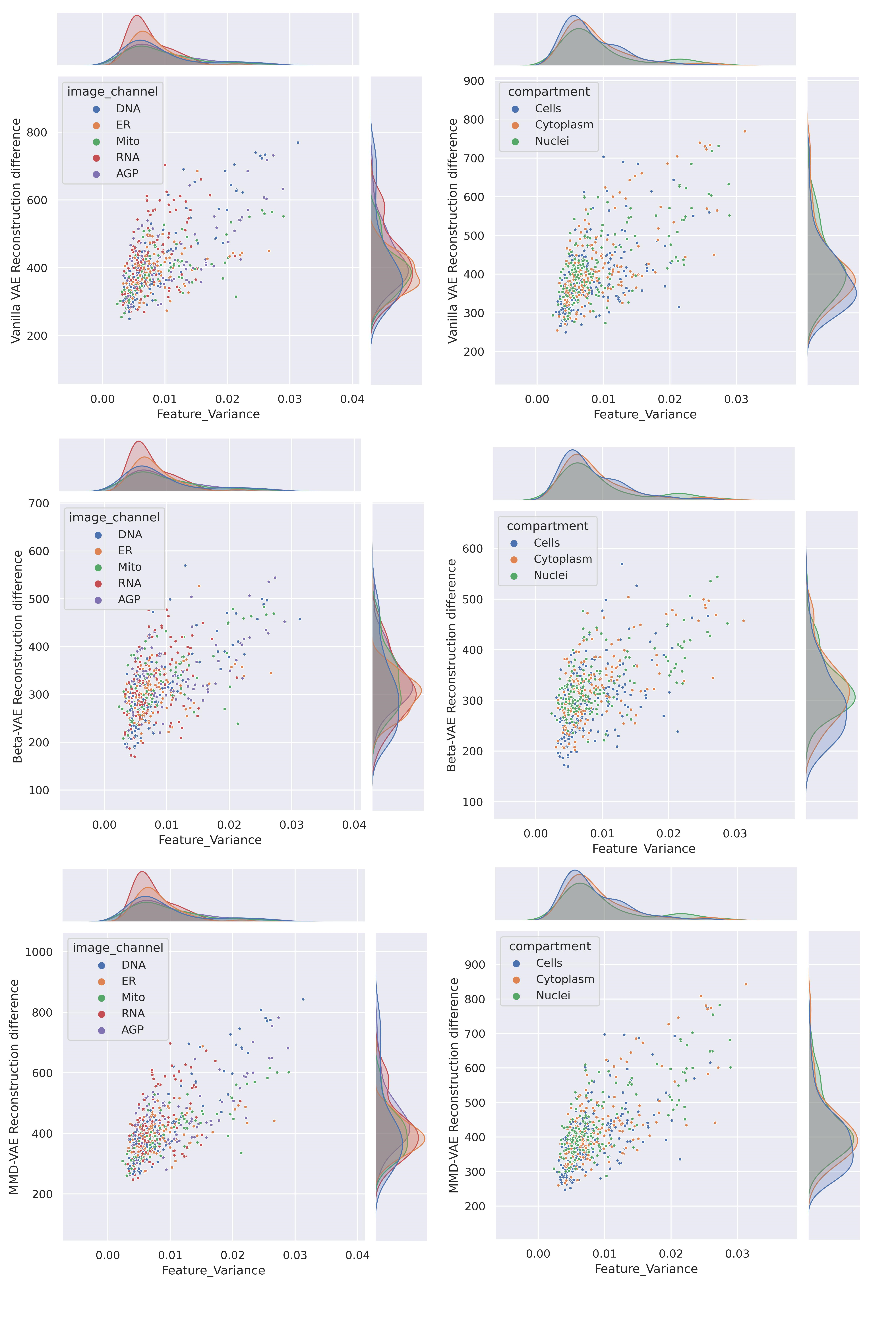

Supplement: S6 Fig — We stratified feature categories by image channel and compartment. (TIFF) [file pcbi.1009888.s006.tiff]

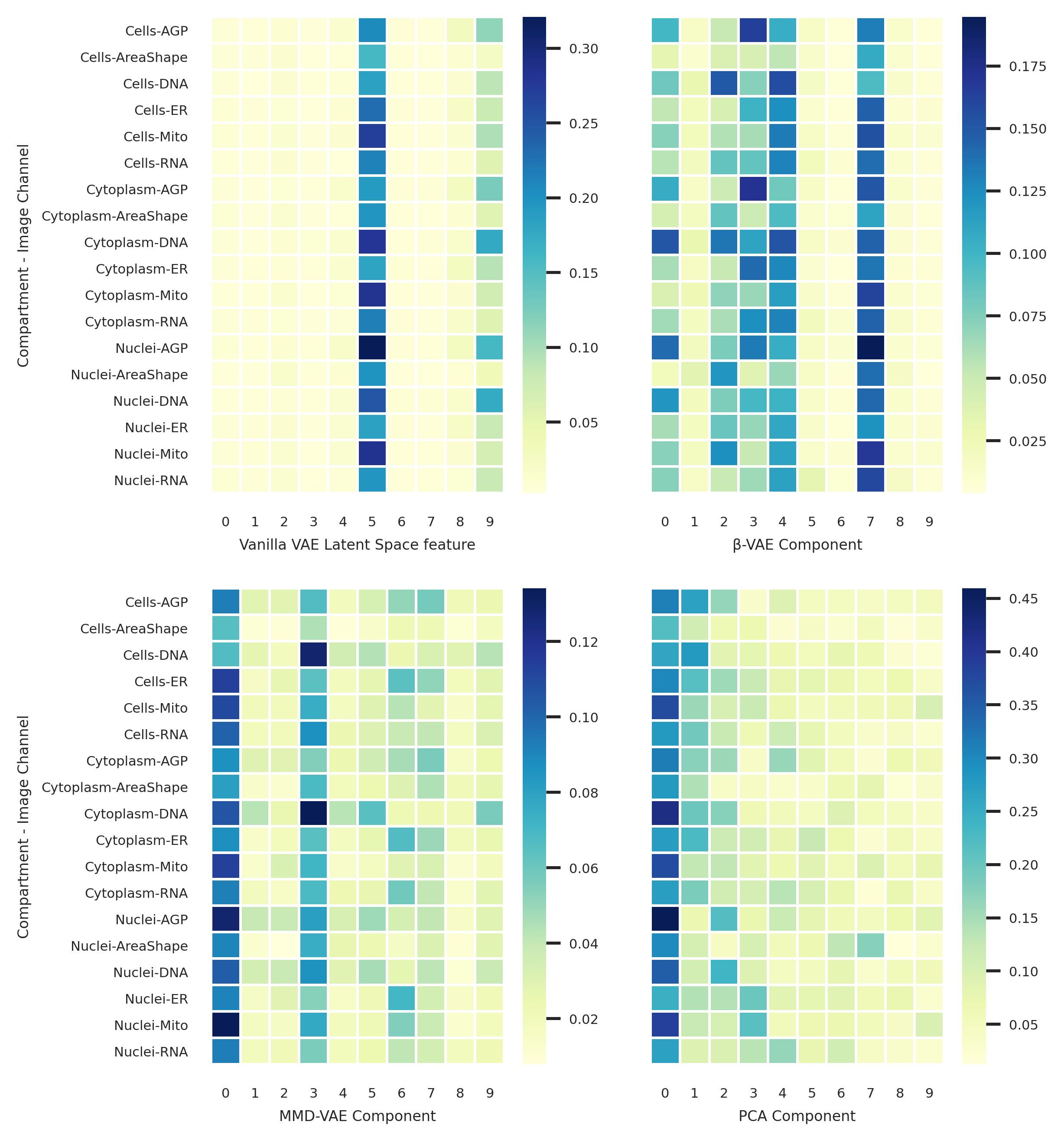

Supplement: S7 Fig — In the training process, VAEs learn latent space embeddings that represent the input features. Here, we systematically modify each latent space feature in isolation (by simulating each latent feature +/- 3 standard deviations, passing through the decoder, and subtracting the original data; see methods for complete details). Each square represents the mean difference between the reconstructed extreme latent feature simulations. The CellProfiler features represent three different cell compartments by five imaging channels plus AreaShape features. Each latent feature across VAE architectures captures a different combination of CellProfiler features. We show heatmaps for all three VAE variants (Vanilla VAE, β-VAE, and MMD-VAE) and the first 10 components of the PCA model. (TIFF) [file pcbi.1009888.s007.tiff]

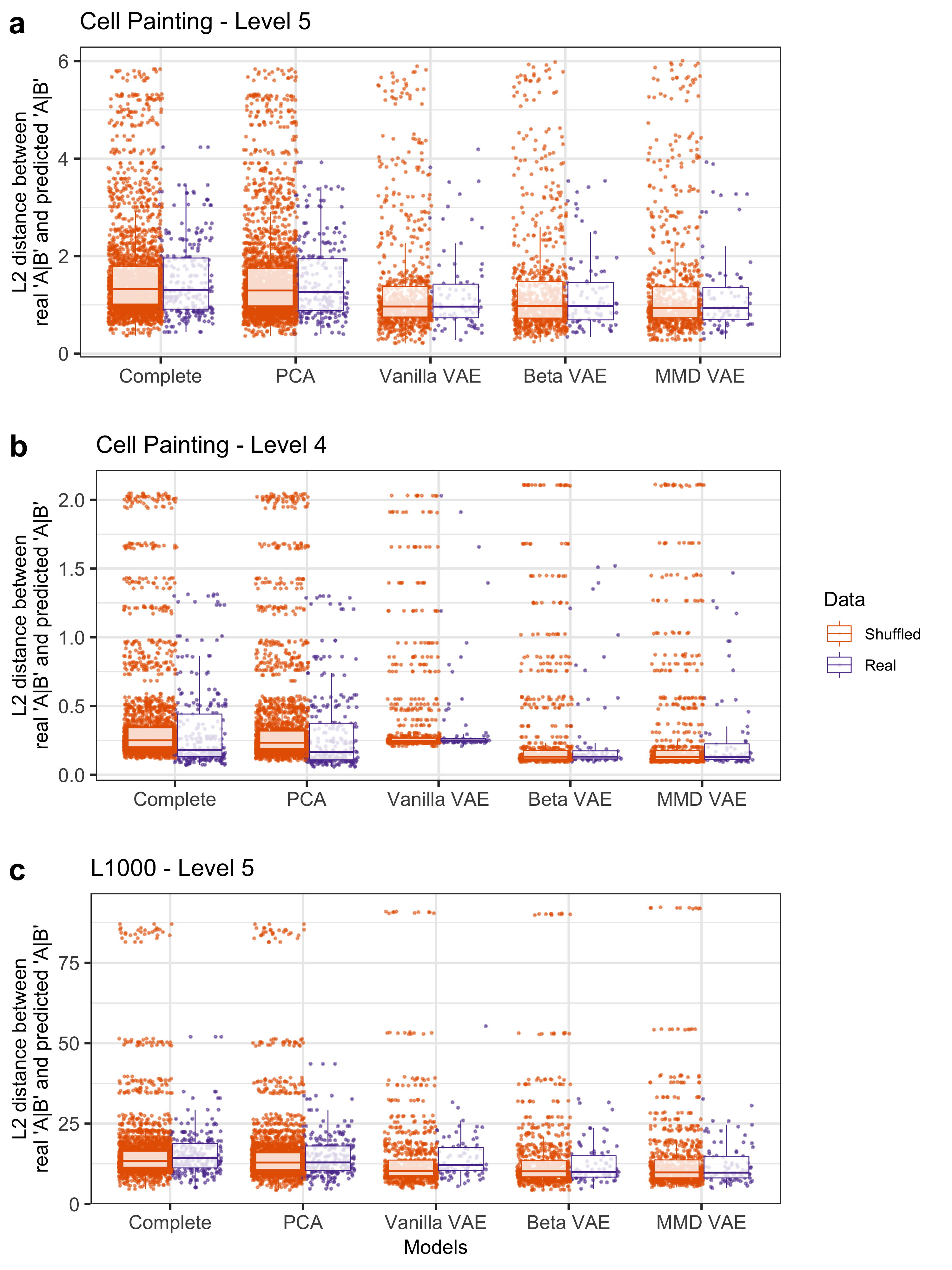

Supplement: S8 Fig — We performed latent space arithmetic to predict cell states of polypharmacological compounds. This allowed us to calculate a L2 distance between predicted and real corresponding to each MOA for both shuffled and real data. We generated distributions for all three datasets and all three VAE variants. (TIFF) [file pcbi.1009888.s008.tiff]

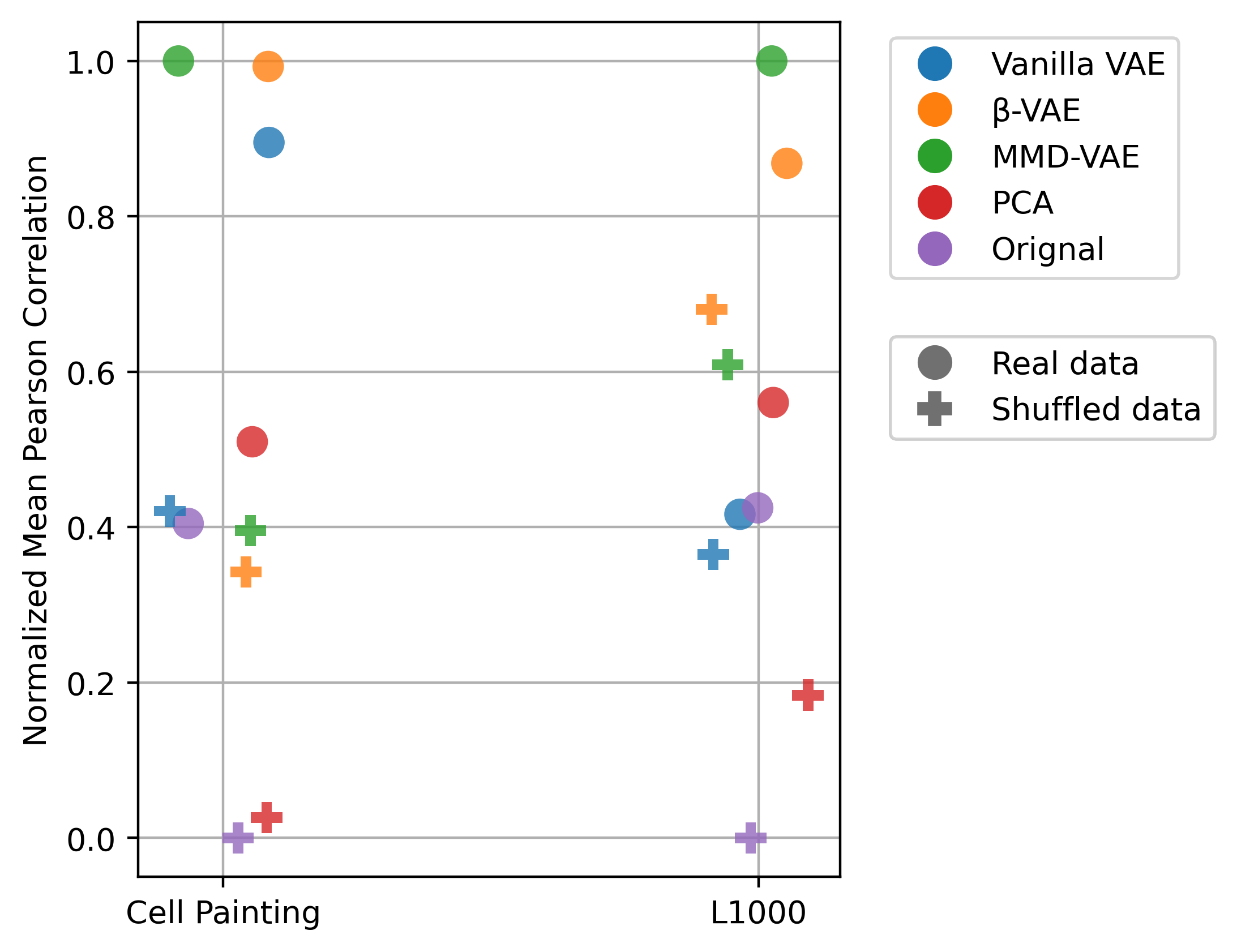

Supplement: S9 Fig — Mean Pearson correlation (higher is better) between real and predicted profiles annotated with known polypharmacology (“A ∩ B”) mechanisms of action (MOAs) for three different VAE architectures, PCA, and original input space. We used level 5 Cell Painting input data for LSA predictions. (TIFF) [file pcbi.1009888.s009.tiff]

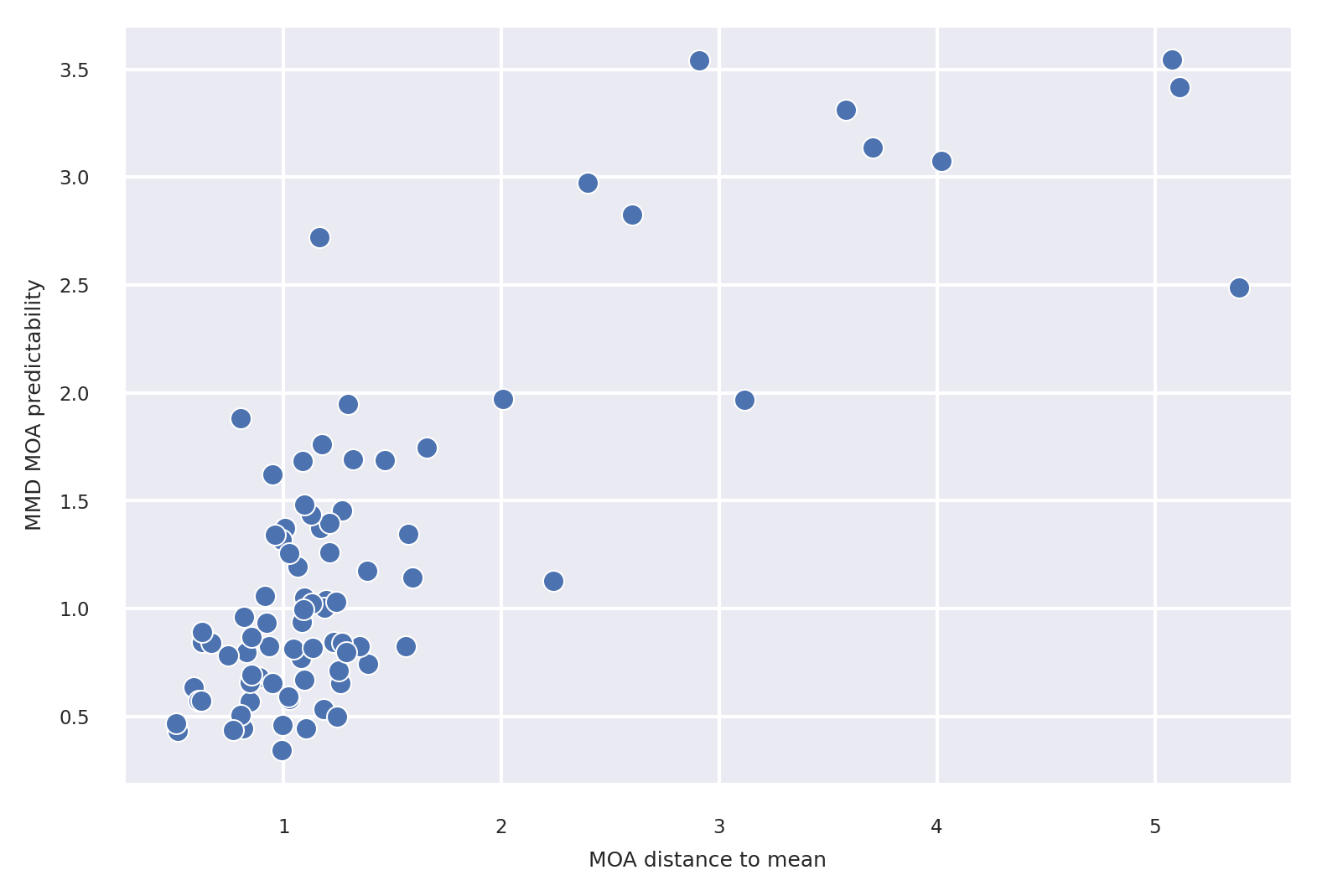

Supplement: S10 Fig — Scatter Plot to visualize the relationship between MOA predictability (- log p value) and the distance between that MOA and the mean Cell Painting feature values in Cell Painting level 5 MMD-VAE. Higher values on the Y axis indicate better predictability, and higher values on the X axis indicate L2 distances to the mean of all profiles together. (TIFF) [file pcbi.1009888.s010.tiff]

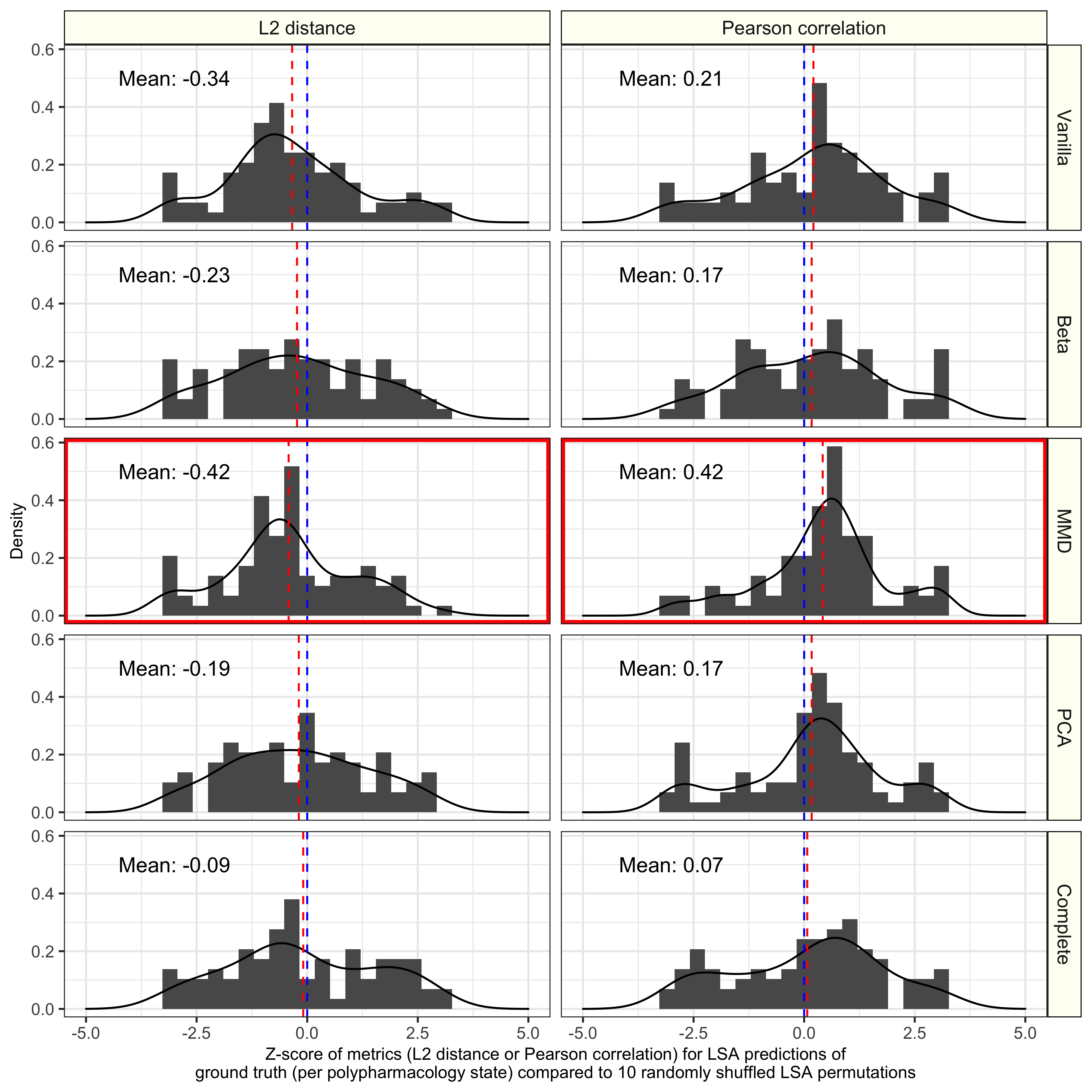

Supplement: S11 Fig — The blue line is centered at 0. All MOAs to the left of the blue line for the L2 distance graph are predicted better than random, and all MOAs to the right of the blue line in the Pearson correlation graph are predicted better than random. The red line indicates the mean of all the z-scores, so a lower mean for the L2 distance is better, and higher mean for Pearson correlation is better. (TIFF) [file pcbi.1009888.s011.tiff]

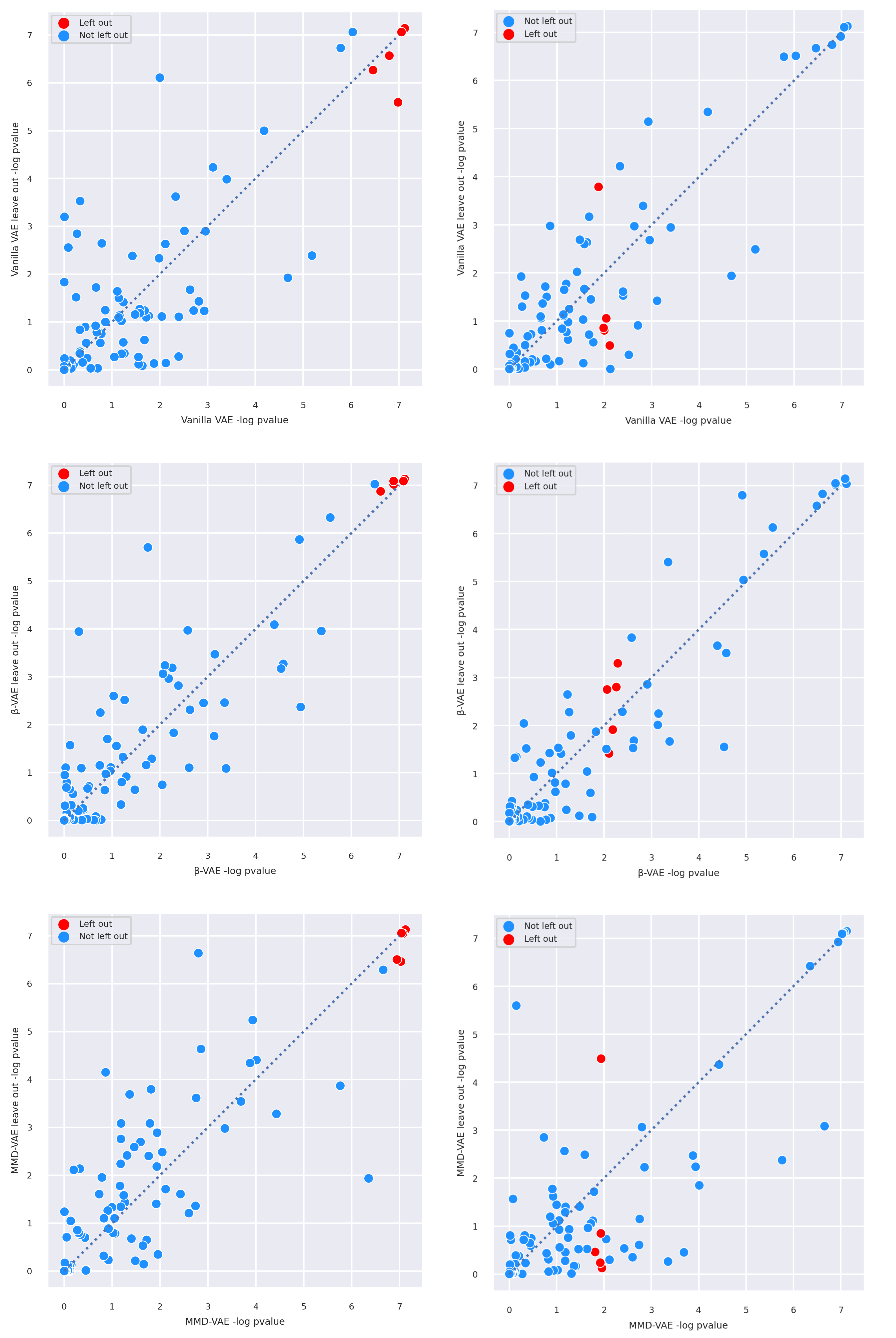

Supplement: S12 Fig — Original polypharmacology MOA prediction latent space performance compared to performance after retraining the VAEs with the top five MOA combinations left out. The axes represent -log10 p value of the L2 distances between real polypharmacology cell states and shuffled cell states in the LSA experiment. The red points represent polypharmacology MOAs that we left out from the models on the y axis. (TIFF) [file pcbi.1009888.s012.tiff]

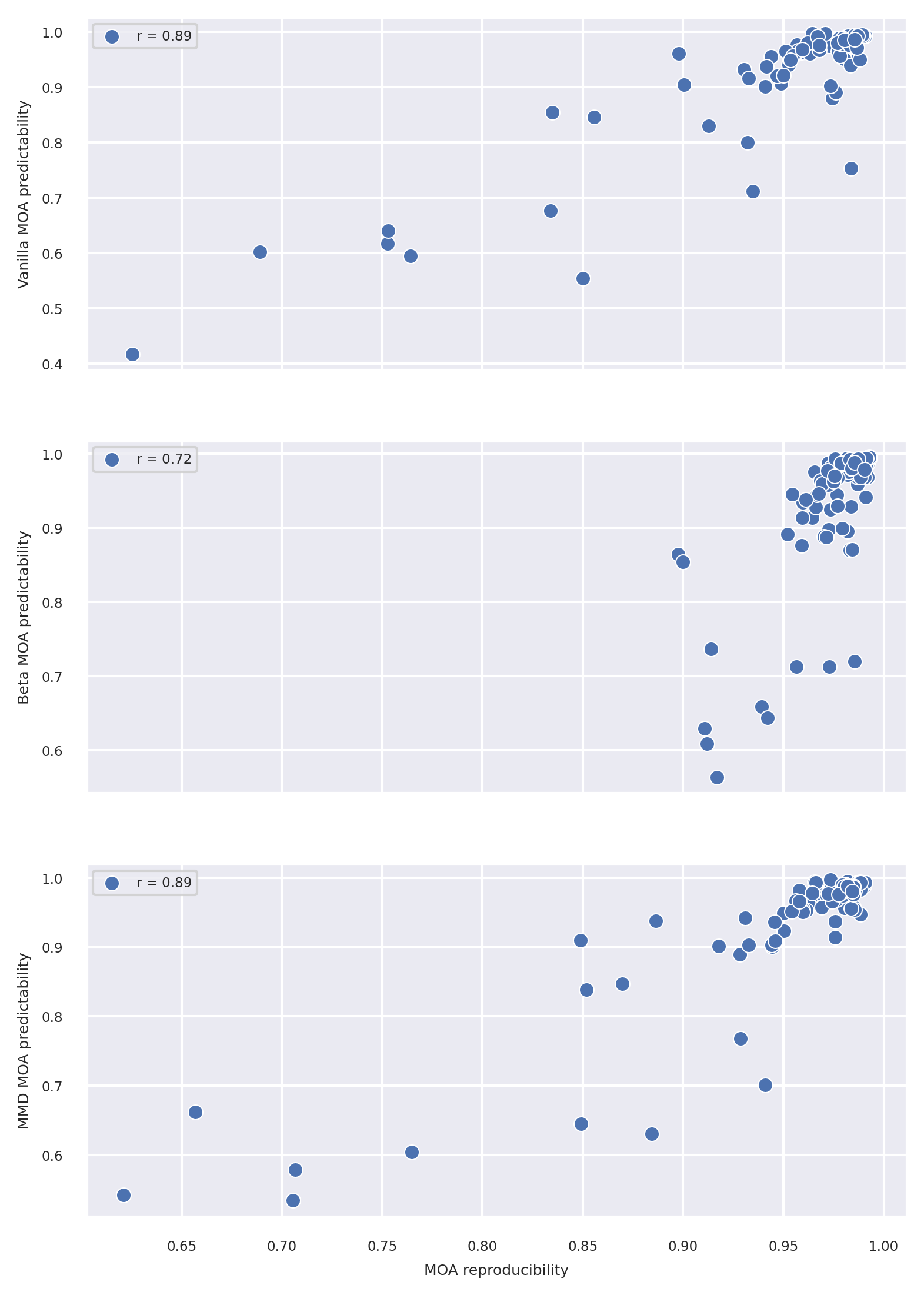

Supplement: S13 Fig — Strong correlation between MOA reproducibility (median pairwise correlation among real and reconstructed MOAs) and MOA predictability (correlation between real and predicted MOA from LSA experiment). (TIFF) [file pcbi.1009888.s013.tiff]

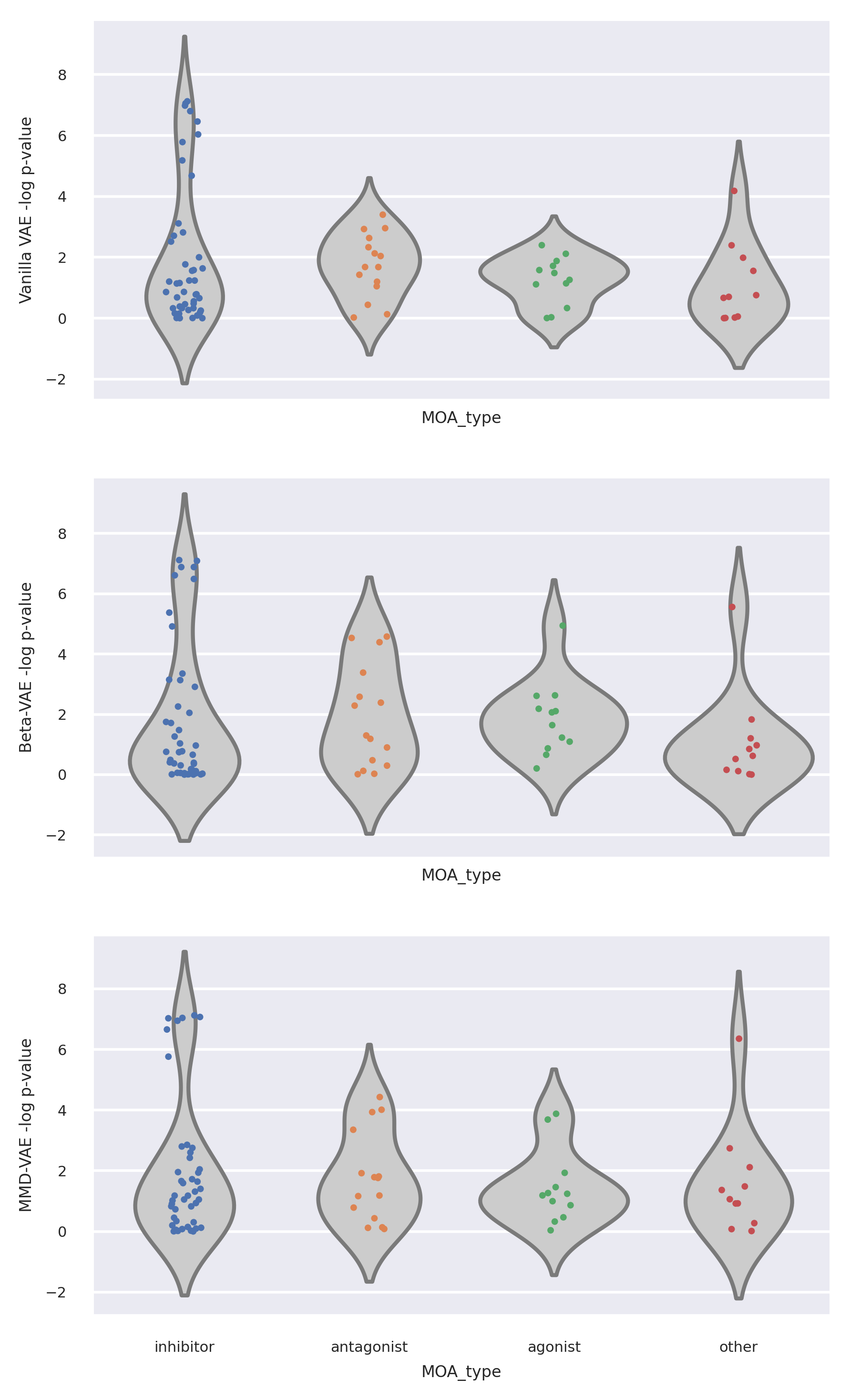

Supplement: S14 Fig — (TIFF) [file pcbi.1009888.s014.tiff]

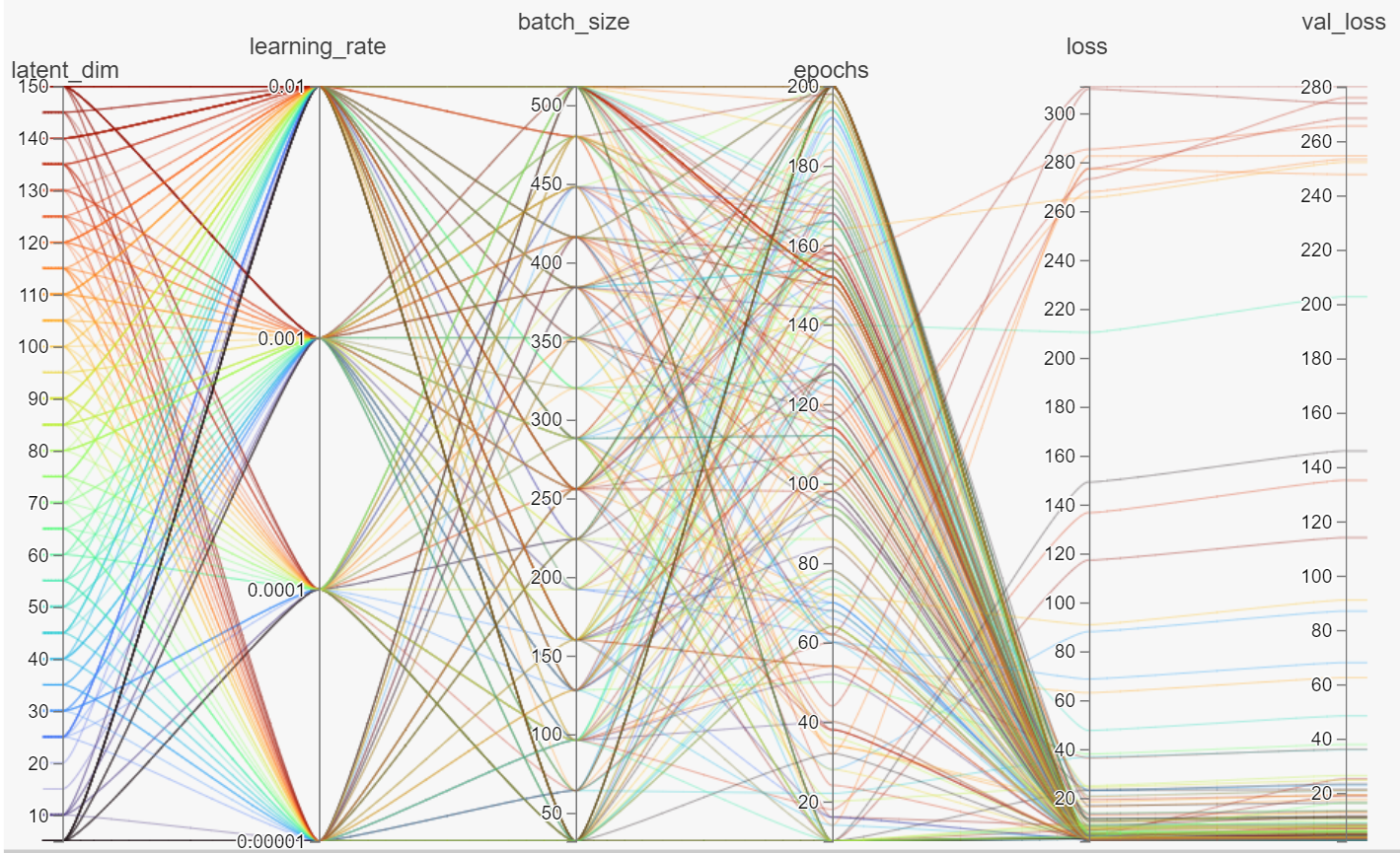

Supplement: S15 Fig — Each line represents a single hyperparameter combination. We show training and validation loss in the last two columns, respectively. (TIFF) [file pcbi.1009888.s015.tiff]
