# Supplementary material for: Predicting drug polypharmacology from cell morphology readouts using variational autoencoder latent space arithmetic
Source: PLoS Comput Biol. 2022 Feb 25;18(2):e1009888. doi: 10.1371/journal.pcbi.1009888 (PMC8906577; doi:10.1371/journal.pcbi.1009888)
Supplement: Attachment — Submitted filename: 120132_1_rebuttal_2214535_r1wyjx.pdf [file pcbi.1009888.s016.pdf]

Revision Plan

Manuscript number: RC-2021-01041
Corresponding author(s): Gregory P. Way, PhD

1. General Statements

On behalf of the authors, I'd like to thank the Review Commons team for sending our manuscript out for review. I'd also like to thank the three anonymous reviewers for providing valuable feedback that will improve the clarity, focus, and analysis interpretation presented in our manuscript.

To prompt the editorial team, our paper provides two well-controlled innovations:

1. *We are the first to train variational autoencoders (VAEs) on classical image features extracted from Cell Painting images.*

   VAEs are commonplace in, and have contributed major discoveries to, other biomedical data types (e.g. transcriptomics), but they have been underexplored in morphology data. In our paper, we trained and optimized three different VAE variants using Cell Painting readouts and compared these variants against shuffled data, against PCA (a nonlinear dimensionality reduction algorithm commonly used as a VAE control), and against L1000 (mRNA) readouts from the same perturbations. We found that cell morphology VAEs train with different settings than gene expression data, and that they generate interpretable latent spaces that depend on the chosen VAE variant.

2. *We tested special VAE properties to predict polypharmacology cell states in a novel way.*

   Polypharmacology is a major reason why drugs fail to reach the bedside. Off-target effects cause unintended toxicity, and lead to adverse clinical events. In our paper, we used VAE latent space arithmetic (LSA) to predict polypharmacology cell states; in other words, what cells might look like if we perturbed them with a compound that had two mechanisms of action (MOA). We compared our results to shuffled data, PCA, and to LSA performed with VAEs trained using L1000 readouts. We found that cell morphology and gene expression provide complementary information, and that we could predict some polypharmacology cell states robustly, while others were more difficult to predict.

We found value in all of the reviewer comments. We intend to conduct all but four of the proposed analyses to supplement our aforementioned innovations.

In the following revision plan, we include all reviewer comments exactly as they were written. The reviewers often had overlapping suggestions. In these cases, we grouped together similar reviewer comments and responded to them once.

We include three sections: 1) A description of the revisions we plan to conduct in the near future; 2) A description of changes we have already made; and 3) A description and rationale of changes we will not pursue.

Lastly, we would like to highlight that all reviewers provided positive feedback in their reviews. They discussed our paper as "conceptually and technically unique" and were positive about our methods section, stating that we did a "good job making everything available and reproducible". Our methods section is complete, and we provide a fully reproducible and versioned github repository. We will release a second version of our github repository when we complete our revision plan to maintain clarity for our submitted version and the peer-reviewed version.

2. Description of the planned revisions

2.1. Address UMAP interpretability to provide a deeper description of MOA performance

Reviewer 1: Instead of using UMAP embedding, it would be better to compare reconstruction error or show a reconstructed image with the original image to claim that models reliably approximate the underlying morphology data.
Reviewer 1: Rather than just stating that the VAE's did not span the original data distribution and saying beta-VAE performed best by eye, some simple metrics can be drawn to analyze the overlap in data for a more direct and quantified comparison. Researchers should also explain what part of the data is not being captured here. Some analysis of what the original uncaptured UMAP represents is important in understanding the limitations of the VAEs' capacity.
Reviewer 2: The authors compare generation performance based on UMAP. In the UMAP space, data tend to cluster together even though they might be far from each other in the feature space. I would like to see more quantitive metrics on how well these methods capture morphology distributions. You can compute metrics like MMD distance, kullback leibler (KL), earthmoving distance, or a simple classifier trained on actual MoA classes tested on generated data.

We agree with the reviewers that evaluating reconstruction loss in addition to providing the UMAP coordinates would improve understanding of VAE limitations and enable a better comparison of VAE performance. We will analyze reconstruction loss across models and include these data as a new supplementary figure, which will enable direct comparisons across models and across different MOAs.

We also agree that UMAP interpretation can be misleading. While currently state-of-the-art, UMAP has mathematical limitations that prevent interpretation of global data structures. However, there are emerging tools, including a new dimensionality reduction algorithm, called PaCMAP, which aims to preserve both local and global structure (Wang *et al*, 2021). We will explore this tool to determine, both mathematically and empirically, which is most appropriate for

our dataset by cross-referencing the visualization with our added supplementary figure describing per-MOA reconstruction loss.

We would also like to emphasize that we trained our VAEs using CellProfiler readouts from Cell Painting images and not the raw Cell Painting images themselves. As this was one of our primary innovations, this detail is extremely important. Therefore, we have improved clarity and added emphasis to this point in the manuscript introduction and discussion (see section 3).

2.2. More specific comparisons of MOA predictions to shuffled data and improved description of MOA label accuracy

Reviewer 1: It is difficult to know the clear threshold for successful performance is on figures like Figure 7 and SFigure 9, but by and large, it appears that the majority of predicted combination MOAs were not successful. Without the ability to either A) adequately predict most all combinations from individual profiles that were used in training or B) an explanation prior to analysis of which combination will be able to predict, it is difficult to see this method being used since the combinatorial predictions are more likely not informative.
Reviewer 1: The researchers justify the poor performance compared to shuffled data, by saying that A) MOA annotations are noisy and unreliable and B) they MOAs may only manifest in other modalities like what was seen in the L1000 vs morphology predictability. While these might be true, knowing this the researchers should make an effort to clean and de-noise their data and select MOAs that are well-known and reliable, as well as, selecting MOAs for which we have a known morphological or genetic reaction.
Reviewer 3: Figure 6 is missing error bars (standard deviation of the L2 distance) and, as such, is hard to draw conclusions from.

We thank the reviewers for raising this concern. We agree that it is critical, and we appreciate the opportunity to address it.

All three of these comments relate to being unable to draw conclusions from our results when most A∩B predictions appear to have no difference from shuffled controls. Therefore, to address this comment, we will update our LSA evaluation to compare each MOA to a matched set of randomly shuffled data. Specifically, in our existing comparison, we realized a methodological fallacy in how we're displaying these data shuffles. We should be comparing specific MOA combinations to their corresponding shuffled results instead of comparing all to all, which will artificially decrease performance when there are polypharmacology predictions that fail to recapitulate the ground truth cell states.

We have connected with Paul Clemons, the senior director Director of Computational Chemical Biology Research at the Broad Institute of MIT and Harvard, who has informed us that the Drug Repurposing Hub annotations are among the most well documented. Therefore, while we know that biological annotations are often incomplete, our original text overemphasized the amount of

noise contributed by inaccurate labels. We therefore added the following sentence to the discussion to clarify this important point:

> "However, the Drug Repurposing Hub MOA annotations are among the most well-documented resources, so other factors like different dose concentration and non-additive effects contribute to weak LSA performance for some compound combinations (Corsello *et al*, 2017)."

We will also update our supplementary figure to account for specific MOA shuffling and include additional text comparing Cell Painting and L1000 showing which MOAs perform best in which modality.

2.3. More detailed evaluation of MOA performance across drug variance and drug classes

Reviewer 1: With the small number of combinations that are successfully predicted, to build confidence in the performance, it would be necessary to explain the reason for the differences in performance. Further experimentations should be done looking into any relationship between the type of MOAs (and their features) and the resulting A|B predictability. Looking at Figure 7, the top-performing combinations are comprised entirely of inhibitor MOAs. If the noisiness of the data is a factor, there should be some measurable correlation between feature noisiness and variation and the resulting A|B predictability from LSA.

We agree with the reviewer that further experimentation would be helpful to gain confidence in our LSA performance. We plan to perform two different analyses to address this question. First, we will compare profile reproducibility (median pairwise correlations among MOAs) to MOA predictability. This will provide insight to determine the relationship between MOA measurement variance and performance. Second, we will split MOAs by category (e.g. inhibitor, activator) and test if there are significant performance differences between categories across VAE models in both L1000 and Cell Painting data. This will tell us if there are certain trends in the type of MOAs we're able to predict. If there is, this would be useful knowledge since it could suggest that certain types of MOAs are associated with a more consistent cell state.

2.4. Higher confidence in LSA overfitting assessment

Reviewer 1: To show that the methodology works well on unseen data, researchers withheld the top 5 performing A|B MOAs (SFig 9) and showed they were still well predicted. This is not the most compelling demonstration since the data to be held out was selected with bias as the top-performing samples. It would be much more interesting to withhold an MOA that was near or only somewhat above the margin of acceptability and see how many holdouts affected the predictability of those more susceptible data points. From my best interpretation, the hold-out experiment also only held out the combination MOA groups from training. It would be better if

single MOAs (for example A) which were a part of a combination of MOA (A|B) were also held out to see if predictability suffered as a result and if generalizability did extend to cells with unseen MOAs (not just cells which had already highly performing combinations of seen MOAs).

We believe our original analysis was extremely compelling. Even if we removed the top MOAs *from training*, we were still able to capture their combination polypharmacology cell states through LSA. We find this similar to removing all pictures of sunglasses in an image corpus of human faces, but still being able to reliably infer pictures of people wearing sunglasses. Specifically, this tells us that our model is learning some fundamental data generating function that our top performing MOAs tap into regardless of if they are present or not in training.

However, we agree with the reviewer that withholding intermediate-performing MOAs would also be informative, but for a separate reason. Unlike the best predicted MOAs, the intermediate MOAs are likely more susceptible to changes in the training data, so it would be interesting to determine if intermediate MOAs' performance is a result of overfitting instead of truly learning aspects of the data generating function. We plan to perform this new analysis and add the results to Supplementary Figure 8 as a subpanel and add a full description of the approach to the appropriate methods subsection.

2.5. Additional metrics to evaluate LSA predictions to provide more confident interpretation

Reviewer 2: The predictions are evaluated using L2 distances, which I find not that informative. I would like to see other metrics (correlation or L1 or distribution distances in previous comments)

We agree with the reviewer that using more than one metric would be helpful because oftentimes a single metric does not tell a complete story. We will add a panel to the LSA supplementary figure (Supplementary Figure 7), using Pearson correlation instead. While L2 distances will tell us how close predictions are to ground truth, Pearson correlations will tell us how consistent, on average, we are able to predict feature direction.

2.6. Adding a performance-driven feature level analysis to categorize per-feature modeling ability

Reviewer 2: I would like to see feature-level analysis, which features are well predicted and which ones are more challenging to predict?

We agree with the reviewer that feature level analysis would be interesting to study. We believe that understanding which features are easy and hard to model could give insight into why certain MOAs (which could be associated with more signal in certain Cell Painting features) are predicted better than others.

However, we are concerned that it is difficult to have an objective measurement of which features are easier to model because features that have less variation might be easier to model. So, we will analyze the correlation between individual feature reconstruction loss vs. feature variance across profiles. We will color-code the points to represent feature groups or channels. This analysis will not only demonstrate the relationship between feature variance and modeling ability, but also provide insight into the difficulty of modeling individual CellProfiler features.

3. Description of the revisions that have already been incorporated in the transferred manuscript

3.1. Documenting positive feedback as provided by the three reviewers

Reviewer 1: With access to the dataset, the posted GitHub, and documentation in the paper, I believe that the experiments are reproducible.
Reviewer 1: The experiments are adequately replicated statistically for conventions of deep learning.
Reviewer 1: This paper proposes a conceptually and technically unique proposal in terms of application, taking existing technologies of VAEs and LSA and, and as far as I know, uses them in a novel area of application (predicting and simulating combination MOAs for compound treatments). If this work is shown to work more broadly and effectively, is seen through to it completion, and is eventually successfully implemented, it will help to evaluate the effects of drugs used in combination on gene expression and cell morphology. An audience in the realm of biological deep learning applications as well as an audience working in the compound and drug testing would be interested in the results of this paper. Authors successfully place their work within the context of existing literature, referencing the numerous VAE applications that they build off of and fit into the field of (Lafarge et al, 2018; Ternes et al, 2021, etc...), citing the applications of LSA in the computer vision community (Radford et al, 2015, Goldsborough et al, 2017), and discussing the biological context that they are working in (Chandrasekaran et al, 2021).
Reviewer 2: The main novelty of the work is applying VAEs on cell painting data to predict drug perturbations. The final use case could be guiding experimental design by predicting unseen data. However, the authors do not show such an example and use case which is understandable due to the need for doing further experiments to validate computational results and maybe not the main focus of this paper. The authors did a good job of citing existing methods and relevant. The potential audience could be the computational biology and applied machine learning community.
Reviewer 3: The manuscript is beautifully written in a crystal clear manner. The authors have made a visible effort towards making their work understandable. The methods section is clear and comprehensive. All experiments are rigorously conducted and the validation procedures are sound. The conclusions of the paper are convincing and most of them are well supported by the

data. Both the data and the code required to reproduce this work are freely available. Overall, the article is of high quality and relevance to several scientific communities.

We thank the reviewers for their encouraging remarks and overall positive sentiment. As early-career researchers, we feel empowered by these words.

3.2. Moved Figure 2 to supplement and removed Figure 5

Reviewer 1: Fig 2 is not informative so it can go to supplementary.
Reviewer 2: I liked the paper's GitHub repo, the authors did a good job making everything available and reproducible. As a suggestion, you can move the learning curves in two the sup figures cause they might not be the most exciting piece of info for the non-technical reader.
Reviewer 3: I would suggest removing Figure 5 (or moving it to the supplementary) as it revisits the content of Figure 1 and does not bring much extra information.

We agree that Figure 2 might not be informative to a non-technical reader, so we have accepted this suggestion by both reviewers 1 and 2, and we have moved Figure 2 to supplementary.

We agree with the reviewer and have removed Figure 5.

3.3. Clarified our data source as CellProfiler readouts, not raw Cell Painting images

Reviewer 1: In Fig 4, it would be useful to show a few sample representative images with respect to CellProfiler feature groups.
Reviewer 1: Figure 6, what does it means original input space? Does it mean raw pixel image? As researchers extracted CellProfiler feature groups already, it would be interesting to compare mean L2 distance based on CellProfiler features so that whether VAE improves performance or not (compared to handcrafted features) as a baseline.
Reviewer 3: While what "morphological readouts" concretely mean becomes clearer later on in the paper, it would be useful to give a couple of examples early on when introducing the considered datasets.

We thank the reviewer for these suggestions, which bring to light a common source of confusion, which we must alleviate. We are working with CellProfiler readouts (features extracted using classical algorithms) of the Cell Painting images and not the images themselves. We have made several edits throughout the manuscript to improve clarity and remove this confusion, including the introduction, in which we clearly state our model input data:

> “Because of the success of VAEs on these various datasets, we sought to determine if VAEs could also be trained using cell morphology readouts (rather than directly on images), and further, to carry out arithmetic to predict novel treatment outcomes. We derive the cell morphology readouts using CellProfiler (McQuin *et al*, 2018), which

measures the size, structure, texture, and intensity of cells, and use these readouts to train all models."

This decision comes with tradeoffs: The benefit of using CellProfiler readouts instead of images is that they are more manageable but we might lose some information. We more thoroughly discuss this important tradeoff in the discussion section:

"We determined that VAEs can be trained on cell morphology readouts rather than directly using the cell images from which they were derived. This decision comes with various trade-offs. Compared to cell images, cell morphology readouts as extracted by image analysis tools (e.g. CellProfiler) are a more manageable data type; the data are smaller, easier to distribute, substantially less expensive to analyze and store, and faster to train (McQuin *et al*, 2018). However, it is likely some biological information is lost, because these tools might fail to measure all morphology signals. The so-called image-based profiling pipeline also loses information, by nature of aggregating inherently single-cell data to bulk consensus signatures (Caicedo *et al*, 2017)."

3.4. Clarified future directions to infer cell health readouts from simulated polypharmacology cell states

Reviewer 1: Authors also make the claim that they can infer toxicity and simulate the mechanism of how two compounds might react. This is a claim that would not be supported even if the method were able to successfully predict morphology or gene profiles. Drug interaction and toxicity are quite complex and goes beyond just morphology and expression. VAEs predicting a small set of features would not be able to capture information beyond the readouts, especially when dealing with potentially unseen compounds for which toxicity is not yet known. For example, two compounds might produce a morphology that appears similar to other safe compounds but has other factors that contribute to toxicity. Further, here they show no evidence of toxicity or interaction analysis.

The reviewer is correct that such a claim is unsupported by our research. Our message was actually that inferring toxicity could be a potential future application of our work. Specifically, for example, we can apply orthogonal models of cell toxicity that we previously derived using other data (Way *et al*, 2021a) to our inferred polypharmacology cell states. We thank this reviewer for noticing our lack of clarity, and we have made changes in the discussion to make it clear that inferring toxicity is something we may do in the future and is not something that is discussed in the manuscript:

"In the future, by predicting cell states of inferred polypharmacology, we can also infer toxicity using orthogonal models (e.g. Way et al. 2021) and simulate the mechanisms of how two compounds might interact."

3.5. Clarified our method of splitting data, and noting how a future analysis will answer overfitting extent

Reviewer 2: Could authors outline detailed data splits? Which MoA are in train and which are held out from training? As I understood, there were samples from MoAs that were supposed to be predicted in the calculation of LSA? Generally, the predicted MoA should not be seen during training and not in LSA calculation.

We now more explicitly detail how we split our data in the methods:

> "As input into our machine learning models, we split the data into an 80% training, 10% validation, and 10% test set, stratified by plate for Cell Painting and stratified by cell line for L1000. In effect, this procedure evenly distributes compounds and MOAs across data splits."

We also thank the reviewer for this comment, because they express an important concern about making sure that we are not overfitting to the data. We have explained in the manuscript that because of lack of data, MOAs were repeated in training and LSA. However, we believe overfitting is not playing a large role in model performance. Through our hold 5 out experiment, we are able to show that our models are able to predict the same MOAs irrespective of whether they were in the training data, indicating that we did not overfit to the distribution of certain MOAs.

Reviewer 1 also suggested that we do the hold 5 out experiment on A∩Bs that were barely predicted. After we do that, we will explicitly demonstrate the extent of overfitting.

3.6. Introduced acronyms when they first appear in the manuscript

Reviewer 3: The Kullback-Leibler divergence is properly introduced in the methods part, but not at all in the introduction (it directly appears as "the KL divergence"). To enhance readability, it would be better to fully spell it before using the acronym, and maybe give a one-sentence intuition of what it is about before pointing out to the methods part for more details.

We thank the reviewer for bringing this to our attention. We have carefully reviewed the entire manuscript and have corrected such instances of clear introductions to acronyms.

3.7. Fixed minor text changes

Reviewer 3: In Figure 1, I would recommend changing "compression algorithms" to "dimension reduction algorithm" or "embedding algorithm". In a compression setting, I would expect the focus to be on the number of bits of information each method requires (or the dimension of the resulting embedding) to encode the data while guaranteeing a certain quality threshold. This is

obviously not the case here as the dimension of the embedding is fixed and the focus is on exploring how the embedding is constructed (eg how much it decorrelates the different features, etc) - which may be misleading.
Reviewer 3: I recommend using "A n B" or "A & B" or "(A, B)" to denote the combination of two independent modes of action A and B. The current notation "A | B" overloads the statistical "A given B" which appears in the VAE loss and is therefore misleading.

We agree with the reviewer, and aim to minimize all sources of potential confusion. We have made the change in the figure.

We also agree that our current notation can be confusing. We have updated all instances of “A|B” with “A ∩ B”.

3.8. Added hypothesis of MMD-VAE oscillations to supplementary figure legend

Reviewer 3: Do the authors have a hypothesis of what may be causing MMD-VAE to oscillate during validation when data are shuffled? This seems to be the case on two of the three considered datasets (Figure 2 and SuppFigure 1) and is not observed for the other models. Including a few sentences on that in the text would be interesting.

We believe a big reason for this is because of the fact that the optimal MMD-VAE had a much higher regularization term, which puts a greater emphasis on forming normal latent distributions, than the optimal Beta or Vanilla VAE. Forcing the VAE to encode a shuffled distribution into a normally distributed latent distribution would be difficult to do consistently across different randomly shuffled data subsets, and therefore might cause oscillations in the training curve across epochs when the penalty for that term is high. As these observations may be interesting to a certain population of readers, we have incorporated this explanation into the supplementary figure legend (which is where this figure is shown):

> “Forcing the VAE to consistently encode a shuffled distribution into a normally distributed latent distribution would be difficult, and therefore might cause oscillations in the training curve across epochs.”

3.9. Explained our selection of VAE variants

Reviewer 3: The different types of considered VAE and their differences are very clearly introduced. It may however be good to motivate a bit more the focus on beta-VAE and MMD-VAE among all the possible VAE models. This is partly done through examples in the second paragraph of page 2, but could be elaborated further.

We thank the author for their encouraging remarks. We have made edits to the manuscript’s introduction, explaining why we chose these two variants out of all the possible choices:

> "We trained vanilla-VAEs, β-VAEs, and MMD-VAEs only, and not other VAE variants and other generative model architectures, such as generative adversarial networks (GANs), because these VAE variants are known to facilitate latent space interpretability."

4. Description of analyses that authors prefer not to carry out

4.1. We will not explore additional latent space dimensions in more detail, as this is out of scope

Reviewer 1: As both reconstructed and simulated data did not span the full original data distribution, it might be better to look at reconstruction error and increase the dimension of latent space.

We thank the reviewer for bringing up this important point. Our VAE loss function consists of the sum of reconstruction error and some form of KL divergence. Specifically, this reviewer is suggesting that if we only minimize reconstruction error (or focus more on reconstruction over KLD by lowering beta), a higher latent dimension would result in better overall reconstruction. This is true, but doing so would have negative consequences. While we would perhaps get the UMAPs to show the full data distribution, the UMAPs are not our focus; predicting polypharmacology through LSA is. We found that when we have a higher focus on the reconstruction term, we have more feature entanglement, as indicated by lower performance when simulating data and overlapping feature contribution per latent feature. The fact that simulating data would logically require less disentanglement than performing LSA shows that we require higher regularization (and hence lower focus on reconstruction) than the one we got from simulating data.

Essentially, while the reviewer's comments would improve reconstruction and allow us to improve the UMAPs, doing so would likely worsen LSA performance, which is the main focus of the project. Also, increasing the latent dimension without changing beta would likely have caused little to no change because since beta is encouraging disentanglement, it would cause the newly added dimension to have little variation and encode little new information that wasn't already encoded before.

We have also previously explored the concept of toggling the latent dimensions in a separate project (Way *et al*, 2020). We are very interested in this area of research in general, and any additional analyses (beyond hyperparameter optimization) deserves a much deeper dive than what we can provide in this paper.

Lastly, we intend to include a deeper description and analysis of reconstruction loss across models, datasets, and MOAs as was suggested by a previous reviewer comment (see section 2.1 above)

4.2. We will not review Gaussian distribution assumptions of the VAE as we feel it is not informative

Reviewer 1: By looking at SFigure 6, I am wondering whether latent distribution actually met gaussian distribution (assumption of VAE). It may show skew distribution as some of latent features shows low contribution.

This reviewer's comment is interesting, but we do not believe it would change the findings of our study. Suppose we find that the latent dimensions aren't normally distributed. This wouldn't change much; a gaussian distribution isn't the most critical to perform LSA. We need the latent code to be disentangled, but having normally distributed latent features doesn't necessarily mean that we have good disentanglement (see https://towardsdatascience.com/what-a-disentangled-net-we-weave-representation-learning-in-vaes-pt-1-9e5dbc205bd1)

4.3. In this paper, we will not train or compare conditional VAEs nor cycle GANs

Reviewer 2: While authors provided a comparison between vanilla VAE and MMD-VAE, B-VEA, there are other methods capable of doing similar tasks (data simulation, counterfactual predictions), I would like to see a comparison with those methods such as conditional VAE(https://papers.nips.cc/paper/2015/hash/8d55a249e6baa5c06772297520da2051-Abstract.html, CVAE + MMD : https://academic.oup.com/bioinformatics/article/36/Supplement_2/i610/6055927?login=true) or cycle GANs(https://arxiv.org/abs/1703.10593).

While such comparisons would be interesting, they are not the main focus of the manuscript, which is to benchmark the use of VAEs in cell morphology readouts and to predict polypharmacology.

We think that CVAE would not be appropriate for our study. In a CVAE, the encoder and decoder are both conditioned to some variable. In our situation where we are predicting the cell states of different MOAs, it would make most sense to condition on the MOA. However, because we're using the MOA labels in our LSA experiment, conditioning on them is likely to bias our results and not be effective for MOAs outside the conditioning.

For cycle GANs, we have found that training using these data, in a separate study in our lab, is extremely difficult. Our lab has not published this yet, but once we are able to better understand cycleGAN behavior in these data, it will require a separate paper in which we compare performance and dissect model properties in much greater detail.

Nevertheless, we have added citations to multi-modal approaches like cycle GANs (see section 4.4) as they will point a reader to useful resources for future directions.

4.4. We will not be comparing with multi-modal integration, but we clarified our focus on Cell Painting VAE novelty and added multi-modal citations

Reviewer 1: Researchers found that the optimal VAE architectures were very different between morphology and gene expression, suggesting that the lessons learned training gene expression VAEs might not necessarily translate to morphology. It would be interesting to compare the result with multimodal integration as baseline (i.e., Seurat).

Our focus in this paper was to train and benchmark different variational autoencoder (VAE) architectures using Cell Painting data and to demonstrate an important, unsolved application in predicting polypharmacology that we show is now possible for a subset of compounds. It was a natural and useful extension to compare Cell Painting VAE performance with L1000 VAE performance especially since our data set contained equivalent drug perturbations. We feel that any extension including multi-modal data integration will distract focus away from the Cell Painting VAE novelty, and requires a much deeper dive beyond scope of our current manuscript.

Additionally, there have been other, more in-depth and very recent multi-modal data integration efforts using the same or similar datasets (Caicedo *et al*, 2021; Haghighi *et al*, 2021). In a separate paper that we just recently submitted, we also dive much deeper to answer the question of how the two modalities complement one another in various ways and for various tasks (Way *et al*, 2021b). These two papers already provide a deeper and more informative exploration of Cell Painting and L1000 data integration.

Therefore, because multi-modal data integration, while certainly interesting, will distract from the Cell Painting VAE novelty and is redundant with other recent publications, we feel it is beyond scope of this current paper.

Nevertheless, multi-modal data integration is important to mention, so we add it to the discussion. Specifically, we discuss how multi-modal data integration might help with predicting polypharmacology in the future and include pertinent citations so that we, or another reader, might be able to follow-up in the future. The new section reads:

> “Because we had access to the same perturbations with L1000 readouts, we were able to compare cell morphology and gene expression results. We found that both models capture complementary information when predicting polypharmacology, which is a similar observation to recent work comparing the different technologies’ information content (Way et al, 2021). We did not explore multi-modal data integration in this project; this has been explored in more detail in other recent publications (Caicedo et al, 2021; Haghighi et al, 2021). However, using multi-modal data integration with models like

CycleGAN or other style transfer algorithms might provide more confidence in our ability to predict polypharmacology in the future (Zhu *et al*, 2017).”

5. References

Caicedo JC, Cooper S, Heigwer F, Warchal S, Qiu P, Molnar C, Vasilevich AS, Barry JD, Bansal HS, Kraus O, *et al* (2017) Data-analysis strategies for image-based cell profiling. *Nat Methods* 14: 849–863

Caicedo JC, Moshkov N, Becker T, Yang K, Horvath P, Dancik V, Wagner BK, Clemons PA, Singh S & Carpenter AE (2021) Predicting compound activity from phenotypic profiles and chemical structures. *bioRxiv*: 2020.12.15.422887

Corsello SM, Bittker JA, Liu Z, Gould J, McCarren P, Hirschman JE, Johnston SE, Vrcic A, Wong B, Khan M, *et al* (2017) The Drug Repurposing Hub: a next-generation drug library and information resource. *Nat Med* 23: 405–408

Haghighi M, Singh S, Caicedo J & Carpenter A (2021) High-Dimensional Gene Expression and Morphology Profiles of Cells across 28,000 Genetic and Chemical Perturbations. *bioRxiv*: 2021.09.08.459417

McQuin C, Goodman A, Chernyshev V, Kamentsky L, Cimini BA, Karhohs KW, Doan M, Ding L, Rafelski SM, Thirstrup D, *et al* (2018) CellProfiler 3.0: Next-generation image processing for biology. *PLoS Biol* 16: e2005970

Wang Y, Huang H, Rudin C & Shaposhnik Y (2021) Understanding how dimension reduction tools work: an empirical approach to deciphering t-SNE, UMAP, TriMAP, and PaCMAP for data visualization. *J Mach Learn Res* 22: 1–73

Way GP, Kost-Alimova M, Shibue T, Harrington WF, Gill S, Piccioni F, Becker T, Shafqat-Abbasi H, Hahn WC, Carpenter AE, *et al* (2021a) Predicting cell health phenotypes using image-based morphology profiling. *Mol Biol Cell* 32: 995–1005

Way GP, Natoli T, Adeboye A, Litichevskiy L, Yang A, Lu X, Caicedo JC, Cimini BA, Karhohs K, Logan DJ, *et al* (2021b) Morphology and gene expression profiling provide complementary information for mapping cell state. *bioRxiv*: 2021.10.21.465335

Way GP, Zietz M, Rubinetti V, Himmelstein DS & Greene CS (2020) Compressing gene expression data using multiple latent space dimensionalities learns complementary biological representations. *Genome Biol* 21: 109

Zhu J-Y, Park T, Isola P & Efros AA (2017) Unpaired Image-to-Image Translation using Cycle-Consistent Adversarial Networks. *arXiv [csCV]*
